# Dynamic Prediction of Reoffending in Individuals Given Community Sentences: Development and Validation of a Novel Risk Monitoring Assessment Tool (OxMore)

**DOI:** 10.1037/lhb0000641

**Published:** 2025-12-04

**Authors:** Denis Yukhnenko, Nigel Blackwood, Paul Lichtenstein, Seena Fazel

**Affiliations:** 1 Department of Psychiatry, University of Oxford; 2 Institute of Psychiatry, Psychology, and Neuroscience, King’s College London; 3 Department of Medical Epidemiology and Biostatistics, Karolinska Institutet

**Keywords:** probation, dynamic risk assessment, psychiatric disorders, community supervision, reoffending prediction

## Abstract

**Objective:**

This study aimed to develop and validate a dynamic risk assessment tool for individuals serving community sentences that accounts for the effects of acute adverse events and desistance from crime.

**Hypotheses:**

Dynamic risk prediction models that incorporate updated data on mental health relapses, incidents of victimization, and desistance from crime will produce more accurate risk stratification for reoffending than models lacking dynamic measurement of risk factors.

**Method:**

We analyzed a national cohort of 59,676 individuals given community sentences in Sweden, of whom 23,879 (45%) had prior psychiatric diagnoses and 18,546 (31%) had substance use disorder diagnoses. Model development tested prespecified criminal history, sociodemographic, and clinical risk factors. Employing landmarking methods for time-to-event data, we modeled the effects of new health care episodes during community supervision, changes in a supervised individual’s circumstances, and the impact of crime desistance. We validated the model in a geographically distinct population.

**Results:**

During follow up, 18,307 (31%) were reconvicted, 4,416 (7%) committed a violent offense, and 5,381 (9%) were hospitalized with a psychiatric diagnosis. The model demonstrated strong calibration and discrimination performance (c-index = 0.74 for violent reoffending, c-index = 0.69 for general reoffending). It also outperformed comparison models that did not incorporate dynamic data. The final model was translated into an online risk calculator (OxMore).

**Conclusions:**

Implementation of dynamic models could lead to more accurate risk stratification for individuals under community supervision, including those with psychiatric and substance use disorders, potentially improving resource allocation, and linkage to interventions that reduce recidivism rates.

To prevent reoffending in sentenced individuals, the criminal justice system assesses the risk of reoffending and aims to implement psychosocial, health-related, and other interventions (Fazel, 2019). The process of assessing criminal recidivism risk typically relies on standardized risk assessment tools (Monahan & Skeem, 2016). These tools generate risk scores or categories that inform both decisions regarding the type, duration, and intensity of supervision and the interventions to be pursued to reduce recidivism risk. Most of these instruments consider a combination of factors that are historical (or static) and those that are modifiable (dynamic).

In many jurisdictions, including the United States, the United Kingdom, and Sweden, community sentences are intended as alternatives to custodial sanctions and are governed by legal frameworks that emphasize proportionality, rehabilitation, and protection of the public. In the context of community sentences, risk assessment is used to support legal decisions about the intensity of supervision, eligibility for treatment programs, and conditions of release or recall. For example, the Swedish Correctional Services Act (§ 10 Kriminalvårdslag, 1974:203) stipulates that probation services must assess the risk of reoffending and develop an individualized supervision plan, which can influence judicial decisions and administrative oversight. Similar frameworks exist in other countries where risk assessments inform legally binding decisions about sentencing conditions, supervisory restrictions, or treatment mandates (Administrative Office of the United States Courts, 2018; Council of Europe, 2017; Monahan & Skeem, 2016). Therefore, inaccurate or outdated risk scores may have legal and ethical consequences, including inappropriate deprivation of liberty or insufficient protection of public safety.

Risk assessment is often conducted using standardized structured or semistructured instruments that assign a risk score or category to an individual based on their characteristics and circumstances, and also highlight potential domains for intervention. In the case of individuals serving community sentences, risk assessment instruments are used to allocate supervision resources and target interventions (Administrative Office of the United States Courts, 2018). In addition to a one-time assessment following sentencing, these instruments are often employed for risk monitoring; that is, to obtain repeated estimates of an individual’s risk score and to measuring their risk factors over time. For example, if an individual changes their employment status from “unemployed” to “employed,” their recidivism risk score would typically decrease. It is usually assumed that the effect of risk factors remains relatively stable over time; therefore, if no change occurs in the risk factors, no change is expected in the risk score. While structured professional judgment instruments such as the Historical Clinical Risk Management-20 Version 3 (Douglas et al., 2014) and the Short-Term Assessment of Risk and Treatability (Webster et al., 2006) explicitly guide practitioners to consider triggers and warning signs in individual cases, these have not been modeled statistically at the group level using robust epidemiological approaches.

If the assumption that risk factor effects remain stable over time does not hold, risk assessment tools relying on it, when used for risk monitoring, will produce inaccurate risk score estimates. Previous research showed that, after committing an offense, an individual’s risk decreased over time and could return to the level of the general population after 5 to 10 years, indicating successful desistance from crime (Hanson, 2018). Given this, over the time horizon of several years, risk assessment evaluations of individuals could accumulate time-dependent bias. To correct this, statistical modeling approaches that account for time-dependent changes in recidivism risk scores should be used when developing risk assessment tools intended for regular risk monitoring. However, to our knowledge, there are no published studies that have developed tools that model changes in individual risk level over time in community sentenced individuals (Fazel et al., 2022).

In addition to risk decay, recent research also supports the proximity hypothesis, which states that dynamic risk assessments are more predictive when conducted closer to the time of the outcome. Prior studies (Davies et al., 2022; Lloyd et al., 2020) found that updated risk scores more accurately predicted imminent recidivism than baseline or averaged assessments. One recent study further demonstrated that the predictive utility of dynamic risk scores declines over time without reassessment (Lee et al., 2024). Their findings also emphasize the need to distinguish between acute and stable dynamic risk factors. Acute risks, such as substance use or emotional instability, degrade more quickly and should be reviewed frequently, whereas stable risks, such as employment status, can be updated less often. Together, this evidence highlights the value of regular reassessment for maintaining predictive accuracy.

Another source of potentially relevant time-dependent information that current risk tools fail to capture are acute/adverse events that lead to sudden changes in individual risk of recidivism. One group of acute events, triggers for crime and violence, have been carefully examined in a large population-based study (Sariaslan et al., 2016). Triggers refer to acute, often adverse, events (such as psychiatric hospitalization, loss of housing, or being a victim of violent crime) that may be associated with a temporary or persistent increase in reoffending risk. These acute events are frequently recorded in administrative records and may precede rapid changes in behavior or be a proxy indicator of underlying severe stress or life difficulties. Previous work has investigated the impact of adverse events, in the form of triggers for crime, and shown that exposure to violence, substance intoxication, unintentional injury, traumatic brain injury (TBI), and self-harm increase the risk of violent crime in the weeks following such events for people with and without mental illness (Sariaslan et al., 2016). Furthermore, TBI was a risk factor for rearrest in people on community sentences (Norman et al., 2023). Commonly used risk assessment instruments do not include these specific triggers associated with risk.

Therefore, the primary aim of this study was to develop and validate a dynamic risk assessment tool for individuals given community sentences, which takes into account the influence of triggers for and desistance from crime. We used data from a large population-based cohort of individuals who had received community sentences in Sweden. The examined variables included risk factors supported by empirical evidence and grounded in theory, relevant to both general and violent reoffending (Fazel et al., 2016; Fazel, Wolf, Vazquez-Montes, et al., 2019; Wolf et al., 2018). Our secondary aim was to assess whether accounting for changes in risk factors over the follow-up period and incorporating desistance would enhance the predictive validity of the developed risk assessment model.

## Method

The present study is a model development and validation study that uses a retrospective cohort design. Transparent reporting of a multivariable prediction model for individual prognosis or diagnosis checklist guidelines for model development and validation were followed (see Appendix A; Collins et al., 2015). This study was approved by the Regional Ethics Committee at the Karolinska Institutet (2013/5:8; Stockholm, Sweden) and carried out according to the relevant guidelines and regulations. Written informed consent from participants was not required as the study was conducted on anonymized routinely collected register data and received ethics approval on this basis.

### Data Sources

We linked the data from several longitudinal, nationwide Swedish registers: the National Crime Register, encompassing data on criminal offenses and convictions dating back to 1973; the National Patient Register, providing psychiatric diagnosis information for inpatient hospitalizations (since 1973) and outpatient care (since 2001); the Migration Register, detailing migration dates to and from Sweden; the Cause of Death Register, with dates and causes of death since 1958; the Multigenerational Register, providing insight into familial relationships for individuals residing in Sweden since 1933; and the Longitudinal Integration Database for Health Insurance and Labor Market studies, supplying annual estimates of income benefit receipt, marital and employment status, and education since 1990. The data linkage utilized a unique personal identifier allocated to all residents and immigrants in Sweden within national registers (Ludvigsson et al., 2009).

### Participants

We included Swedish residents aged 18 and above who had received any community sentence between January 1, 2007, and December 31, 2013. We excluded individuals born before 1958, as they would lack an uninterrupted criminal record in the National Crime Register, given that the age of criminal responsibility is 15, and criminal data were available from 1973 onward. Moreover, individuals who committed an offense before the beginning of the follow-up period but had not been sentenced for it by that time (referred to as pseudoreconviction) were omitted. Individuals whose cases had been appealed or dismissed were also excluded, as their information was not present in the sentencing register.

The community sentences comprised probation with community service, probation with contracted treatment, and conditional sentences mandating community service. These sanctions covered all types of community-based penalties in Sweden, delineated in Chapters 27 and 28 of the Swedish Penal Code (Boijsen & Tallving, 2017), excluding postcustodial supervision and probation concurrent with imprisonment. The initiation of the follow-up period for each individual was determined by the date of receiving a community sentence. In cases where an individual received multiple community sentences during the study period, we randomly selected the index sentence and used its date as the starting point for follow up. This method ensured the inclusion of both individuals undergoing their initial community sentence and those with prior criminal records who had multiple community sentences. As a result, we ensured that the analysis cohort was more comprehensive and representative than the cohort consisting exclusively of individuals serving their first sentence.

### Outcomes and Censoring

We developed separate models for two primary outcomes: the probability of violent reoffending and the probability of general reoffending, each within a 2-year window. General reoffending was defined as the commission of any offense following the index sentence, while violent reoffending included offenses such as homicide, assault, robbery, arson, sexual offenses, illegal threats, or intimidation. Offense dates were obtained from national registers and are recorded retrospectively once established by a court; if no offense date was available, the sentencing date was used as a proxy. Probabilities for both outcomes were estimated dynamically at sentencing and at 36 subsequent monthly landmarks during a 3-year follow-up period. A *landmark* refers to a predefined time point at which a new prediction is generated, using information available up to that time.

Censoring events were death from any cause, permanent emigration from Sweden, and, in the case of violent reoffending, imprisonment for a nonviolent crime. If an individual observation was censored before the end of the 2-year follow up, it was removed from the training and validation data.

### Variable Specification

We categorized all predictor variables used in the analysis into four distinct groups (Appendix B). For the sake of coefficient interpretability, we did not consider interactions among covariates.

Group 1 included sex and previous criminal history covariates measured at the time of sentencing. All of the Group 1 covariates were included in all models by default. Group 2 consisted of covariates measured at baseline but subject to change during the follow-up period, reflecting an individual’s current status. This group included sociodemographic factors (that also included in Group 3), such as civil status, employment, receipt of income support, and housing stability, but measured dynamically and updated at each landmark using the most recent available data. In addition, Group 2 included current age and psychiatric diagnoses recorded prior to a given landmark. The variable “any psychiatric diagnosis/disorder” was defined using *International Statistical Classification of Diseases*, 10th revision diagnostic codes (F00–F99) recorded in the National Patient Register. This included individuals with or without a co-occurring substance use disorder (F10–F19). These covariates were included by default, as they capture time-varying aspects of risk that may influence reoffending trajectories during community supervision. Covariate values were updated every 30 days, starting from their initial values on the first day of the follow-up period, with these time points termed “landmarks” within the survival analysis framework using a landmarking approach (as described below). Since sociodemographic covariates were recorded annually (in November), we carried forward the most recent measurements until the next available record.

Group 3 consisted of covariates measured solely at baseline that were not updated during follow up but were thought, based on prior literature and theoretical relevance, to be associated with the outcomes. These included civil status (coded as single vs. not single, with the latter category including individuals who were married or in a registered partnership), employment status, receipt of income support, unstable housing (defined as more than three address changes within 1 year), and a recorded history of self-harm or suicide attempt. The inclusion of Group 3 covariates in the final models depended on the outcome of the variable selection process outlined below.

Group 4 comprised covariates exclusively measured during the follow-up period and likely associated with reoffending, such as triggers for violent crime (Sariaslan et al., 2016) and psychiatric hospitalizations, serving as a proxy for acute and significant psychiatric symptomatology. We introduced the time-dependent impact of each trigger as three distinct binary variables, representing three hypothetical aspects of a trigger’s influence: the “acute effect,” “short-term effect,” and “residual effect.” The acute effect, indicating a risk surge, was assigned a value of 1 if a trigger event occurred within a week before a specific 30-day covariate update (landmark); otherwise, it was coded as 0. The short-term effect was assigned a value of 1 if a trigger event occurred within a month before the update; otherwise, it was coded as 0. The residual effect was assigned a value of 1 if a trigger event occurred at any time from the beginning of the follow up until a specific update; otherwise, it was coded as 0. Covariates for acute and short-term effects can be understood as modifiers of the residual trigger effect during their corresponding time windows.

Immutable personal characteristics such as sex and age are among the strongest predictors of recidivism and are included in most actuarial risk assessment instruments. In Sweden, the United Kingdom, the United States, and many other countries, their use is legally permissible and routinely incorporated into risk modeling. However, in some jurisdictions, their inclusion may raise legal or constitutional concerns, and their use should therefore be carefully considered in context, and may require adaptations (e.g., in training).

### Handling of the Missing Data

The percentage of missing data for the included covariates varied from 0.1% to 3.2% at the time of sentencing, that is at baseline (Appendix C). We performed imputations for sociodemographic factors both at the baseline and during the follow up. In instances of missing sociodemographic factors during follow up, we utilized baseline values for the initial 3 months of the follow-up period, with 3 months representing the median time from the start of the follow up to the subsequent measurement. For education level, we extended the last recorded measurement until the next available measurement without any time restrictions. Missing values for other sociodemographic records were imputed using an expected-maximization algorithm implemented in the *Amelia* package for R (Honaker et al., 2011), with the time of measurement serving as a cross-sectional time-series indicator. All measured covariates and outcome variables were utilized as predictors for missing data points (Sterne et al., 2009).

Clinical covariates, including triggers, were recorded in the register only if the corresponding event had occurred. Consequently, we assumed complete information about clinical covariates in the data set and no imputation for missing values was deemed necessary.

### Data Splitting

The data set encompassed the entire cohort, which was split into a derivation sample (approximately 80% of the data) used for developing and internally validating the predictive model, and an external validation sample (around 20% of the data). The derivation set was selected from the entire cohort based on the individual’s residential geographical location at the time of sentencing. Regions were primarily defined by the counties of Sweden, identified from the initial two digits of the Swedish Small Area Market Statistics code. Exceptions were made for the municipalities of Gothenburg and Malmö, which were distinct from their corresponding counties, and the Stockholm municipality, which was separated from its county and subdivided into northern and southern parts by associating each Swedish Small Area Market Statistics area with its historical province. These regions were categorized into four groups (Appendix B), serving as proxy indicators of urban/rural status.

This stratified geographic splitting strategy was designed to maximize heterogeneity between the derivation and validation samples while maintaining representativeness within each. By incorporating variation in population density and service infrastructure, it enabled a more rigorous evaluation of the model’s generalizability across different criminal justice settings. The external validation set was geographically distinct and selected randomly, with equal probability, choosing one region from each of the first three groups and proceeding sequentially through the fourth group. This methodology, previously utilized for other evidence-based tools, is recommended to avoid overfitting in the validation sample and to maintain cohort representativeness (Fazel, Wolf, Larsson, et al., 2019).

### Modeling Process

The predictive modeling employed Cox proportional hazards regression with sliding window landmarks (van Houwelingen & Putter, 2011) adjusted for measured covariates. A “landmark” denotes a specific time point at which the values of covariates were updated; in our study, this occurred every 30 days of the follow up. At each landmark, we also recalculated the probability of the outcomes. Consequently, each landmark represented a time point for reevaluating the risk, and for each landmark, a distinct baseline hazard was estimated. In general, the landmark approach involves splitting the follow-up period into a series of overlapping time intervals and fitting separate risk models for each interval, using the information available at that time point. By then combining these models, the approach captures how risk changes over time in response to updated information.

In the analysis, we used 37 landmarks, starting from the commencement of the sentence (Landmark 0) and covering each month of the 3-year follow-up period (Landmarks 1–36). Individual data sets were created for each landmark, comprising only those individuals who were at risk at that specific landmark’s time, along with their corresponding covariate values. All 37 landmark data sets were subsequently consolidated into a single comprehensive landmark superset. The change in baseline hazard from one landmark to another landmark models the effect of desistance from crime since the start of the follow-up period.

To derive estimates of coefficients for the final prediction model, we combined results across multiple imputations using Rubin’s rule (Barnard & Rubin, 1999). Rubin’s rule involves calculating the average of the parameter estimates across all imputed data sets to obtain a pooled point estimate. It then combines the within-imputation variance (reflecting sampling variability in each imputed data set) and the between-imputation variance (reflecting uncertainty due to missing data) to produce a total variance estimate. This approach yields valid standard errors, confidence intervals, and significance tests under the assumption that data are missing at random. By incorporating both sources of uncertainty, Rubin’s rule ensures that statistical inferences appropriately reflect the variability introduced by imputation.

### Variable Selection

The variable selection process for the current model followed the general approach implemented during the development of the OxRec (Fazel et al., 2016), FoVoX (Wolf et al., 2018), and OxMIV (Fazel, Wolf, Larsson, et al., 2019) prediction tools. All variables from Groups 1 and 2 were included in the final model on the basis of theory. To further select predictors from Groups 3 and 4, we employed an automatic backward elimination approach based on combined estimates from all imputed data sets (Wood et al., 2008). We used a *p* value threshold of 0.157, which is equivalent to model selection using Akaike information criterion (Sauerbrei, 1999), for excluding variables, following the convention to ensure model balance and prevent overfitting, as suggested in Heinze and Dunkler (2017).

This approach ensures the face validity of the final model while concurrently permitting the inclusion of additional risk factors if they display an association with reoffending outcomes. The classification of variables into these four groups aims to produce as parsimonious a model as possible (i.e., easier to implement in practice), as long as it maintains acceptable predictive performance.

Several variables were reoperationalized after the first variable selection (see Appendices D and E). The primary dynamic landmark model (DLM) for violent reoffending included: baseline factors (age, sex, and employment), being a victim of a violent assault, TBI or injuries from other causes, any psychiatric hospitalization, substance intoxication, any prior self-harm or suicide attempt. These factors were also included in the final model for general reoffending, although the triggers had different time components. DLM for general reoffending also included the receipt of income support at baseline as a predictor (see Appendix F for DLM formulae, coefficients, and baseline hazards).

### Assessing Model Performance

The models’ discriminative accuracy on the derivation data set was assessed with the optimism-corrected c-index and receiver operating characteristic (ROC) curve analysis. To obtain the c-index corrected for optimism, we implemented bootstrap-based Harrell’s bias correction method (Harrell et al., 1996; Iba et al., 2021). Specifically, the model was refitted on each of 200 bootstrap samples drawn from the derivation set, and its discrimination and calibration were evaluated in both the bootstrap and original samples. The average difference in performance (i.e., optimism) was subtracted from the apparent performance in the original sample to yield optimism-corrected metrics. Final estimates of discrimination and optimism were obtained by pooling results across all iterations, with 95% confidence intervals derived from corresponding quantiles.

Model calibration was evaluated by comparing predicted and observed event probabilities across risk levels and time windows using calibration plots. Prediction error was summarized using the Brier score and the Brier Skill Score was calculated relative to a naïve model that assigned zero reoffending probability to all individuals.

Furthermore, the performance of the final model (DLM) was compared with two other models. First, the baseline model with fixed covariates (fixed baseline model or FBL) was trained using only the information available at the time of the sentence. For this model, the values of covariates were fixed at their baseline level and were not updated during the follow up. Also, the FBL model only incorporated the baseline hazard estimated at time point 0 of the follow up. FBL corresponds to the standard approach to developing a risk prediction model. Second, the landmark model with fixed covariates (fixed landmark model or FLM) was fitted using the information available at all landmark times but without a dynamic update of the covariate values. For this model, the values of covariates were fixed at their baseline values and did not change during the follow up between landmarks. The occurrence of the triggers was additionally not considered. However, this model incorporated separate baseline hazard estimations for each landmark time. FLM corresponds to the basic approach of developing a simple dynamic risk prediction model that incorporates the effect of desistance from crime, modeling the change in baseline hazard while keeping the covariates constant.

### Model Formula

The final prediction model produced individual-level risk scores using the formula

(1) P(reoffending)=1−exp(−BHLM⋅exp(∑β⋅RF)),

where BHLM denotes the baseline hazard at a given landmark, β represents regression coefficients, and RF denotes binary-coded risk factor presence. Model coefficients were derived separately for violent and general reoffending.

### Statistical Software

We extracted and preprocessed data using SAS, Version 9 for Windows (SAS Institute Inc, 2013). The data transformation and the model development were done in RStudio (R Core Team, 2013; RStudio Team, 2020). For the main analyses and modeling, we used the following R packages: dynpred (Putter & Putter, 2011), survival (Therneau, 2021), Amelia (Honaker et al., 2011), and pROC (Robin et al., 2011). The plots were created with the survminer (Kassambara et al., 2017) and ggplot2 packages (Wickham et al., 2016). Some code was adapted from van Houwelingen and Putter (2011) and Hazewinkel (2018). Denis Yukhnenko also wrote custom code for variable selection, optimism-corrected internal validation, out-of-sample prediction for the trained models, and calibration assessment. This was required as many built-in functions of the available packages either do not work with landmark data or work very inefficiently on large data sets. The code is available via GitHub by request (Yukhnenko, 2025).

## Results

The study cohort consisted of 59,676 individuals given community sentences in Sweden between 2007 and 2013 (Table 1). Median follow-up time was 23 months. During follow up, 18,307 (31%) were reconvicted and 4,416 (7%) committed a violent offense. The external validation sample contained 28% of all individuals. The derivation and external validation samples had similar baseline characteristics (Table 1).

The prevalence of individuals who experienced triggers is presented in Appendix G for both derivation and external validation samples. Observed rates for reoffending at 12, 24, and 36 months were similar for derivation and validation samples (see Appendix H).

### Internal Validation

The optimism-corrected c-index for the primary model (DLM) was .74 for 2-year violent reoffending and .69 for 2-year general reoffending, indicating good overall discrimination (Appendix D). In the ROC analysis, DLM also demonstrated good discrimination over time. For violent reoffending, the area under curve (AUC) at the start of a sentence was .76 (95% CI [.75, .77]) and decreased to .75 (95% CI [.72, .77]) in individuals with the offense-free time of 36 months (Appendix Figure I1). For general reoffending, the AUC at the start of a sentence was .75 (95% CI [.74, .75]) and decreased to .67 (95% CI [.65, .69]) in individuals with the offense-free time of 36 months (Appendix Figure I1).

**Figure 1:**
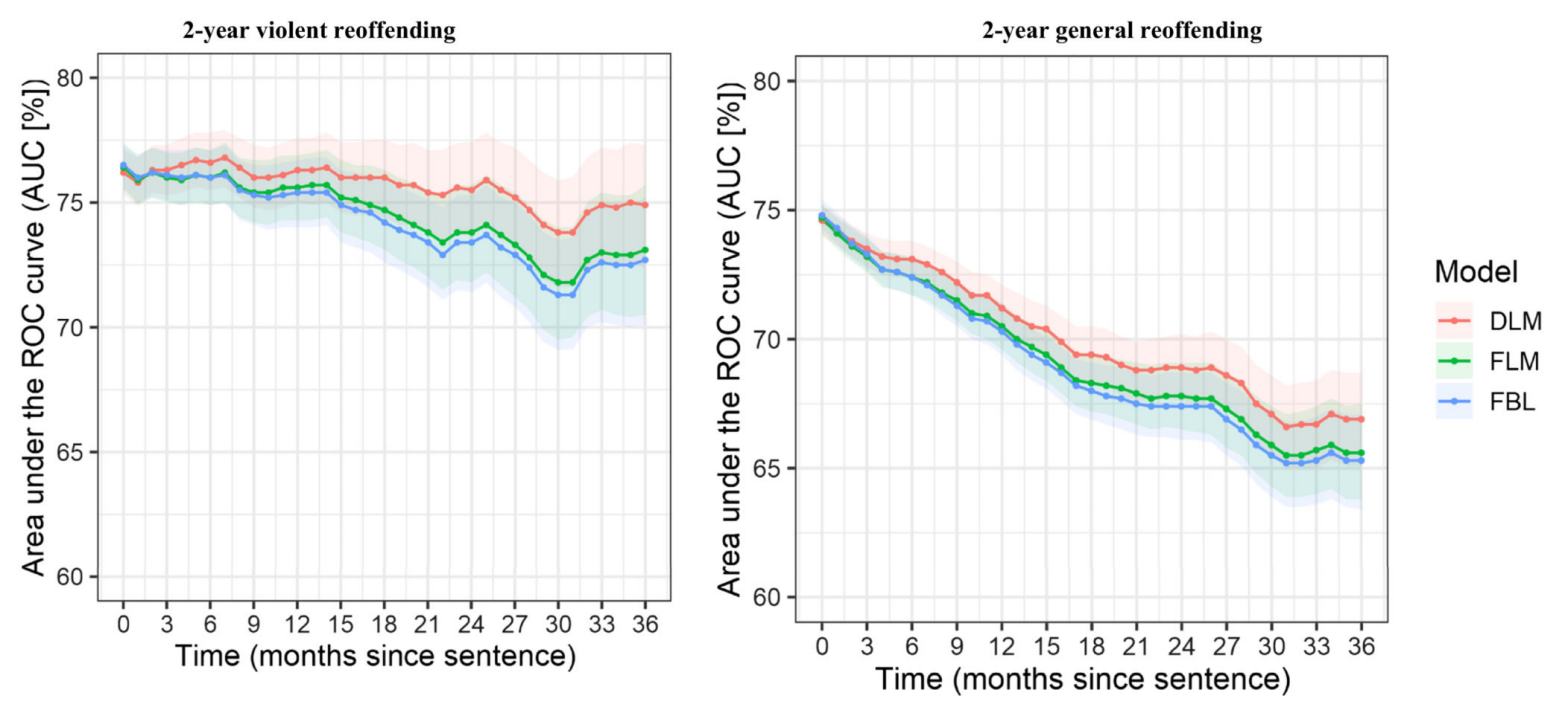
Discrimination Performance of the Prediction Models Over Time in the Internal Validation Data Set *Note*. DLM and FLM account for offense-free time. FBL only uses covariate values at baseline and does not account for offense-free time. Discrimination performance is shown as time-varying AUCs across follow-up for the three models. ROC = receiver operating characteristic; AUC = area under curve; DLM = dynamic landmark model; FLM = landmark model with fixed covariates; FBL = baseline model with fixed covariates. See the online article for the color version of this figure.

DLM also demonstrated moderate prediction error for 2-year violent and general reoffending probabilities as assessed by Brier scores (Figure 1). The performance gain relative to a naïve model, which assigned zero probability of outcomes to every individual, was more apparent for general reoffending. The prediction error also increased over time, approaching the performance level of a naïve model.

### External Validation and Model Comparison

The primary model (DLM) demonstrated good overall discrimination on the external validation data set, with higher mean c-index values for both violent (.75) and general reoffending (.72) compared with the FLM (FLM: .74 and .71, respectively) and the baseline model (FBL: .73 and .69, respectively).

Over time, for violent reoffending, the AUC at the start of a sentence was .76 (95% CI [.74, .78]) and decreased to .71 (95% CI [.67, .76]) in individuals with the offense-free time of 36 months (Appendix I). For general reoffending, the AUC at the start of a sentence was .76 (95% CI [.75, .76]) and decreased to .68 (95% CI [.65, .71]) in individuals with the offense-free time of 36 months (Appendix I). DLM demonstrated marginally better discrimination performance over time for general and violent reoffending compared with the landmark model with fixed covariates (FLM) and the baseline model with fixed covariates (FBL; Figure 1). The point estimates for discrimination performance of FLM and FBL were almost identical. Both models used the covariate values estimated at baseline.

Similar to internal validation performance, DLM demonstrated moderate prediction error for 2-year violent and general reoffending probabilities as assessed by Brier scores (Figure 1). The performance of DLM and FLM was comparable. Both landmark models outperformed FBL, which accumulated prediction error at a much faster rate.

**Figure 2:**
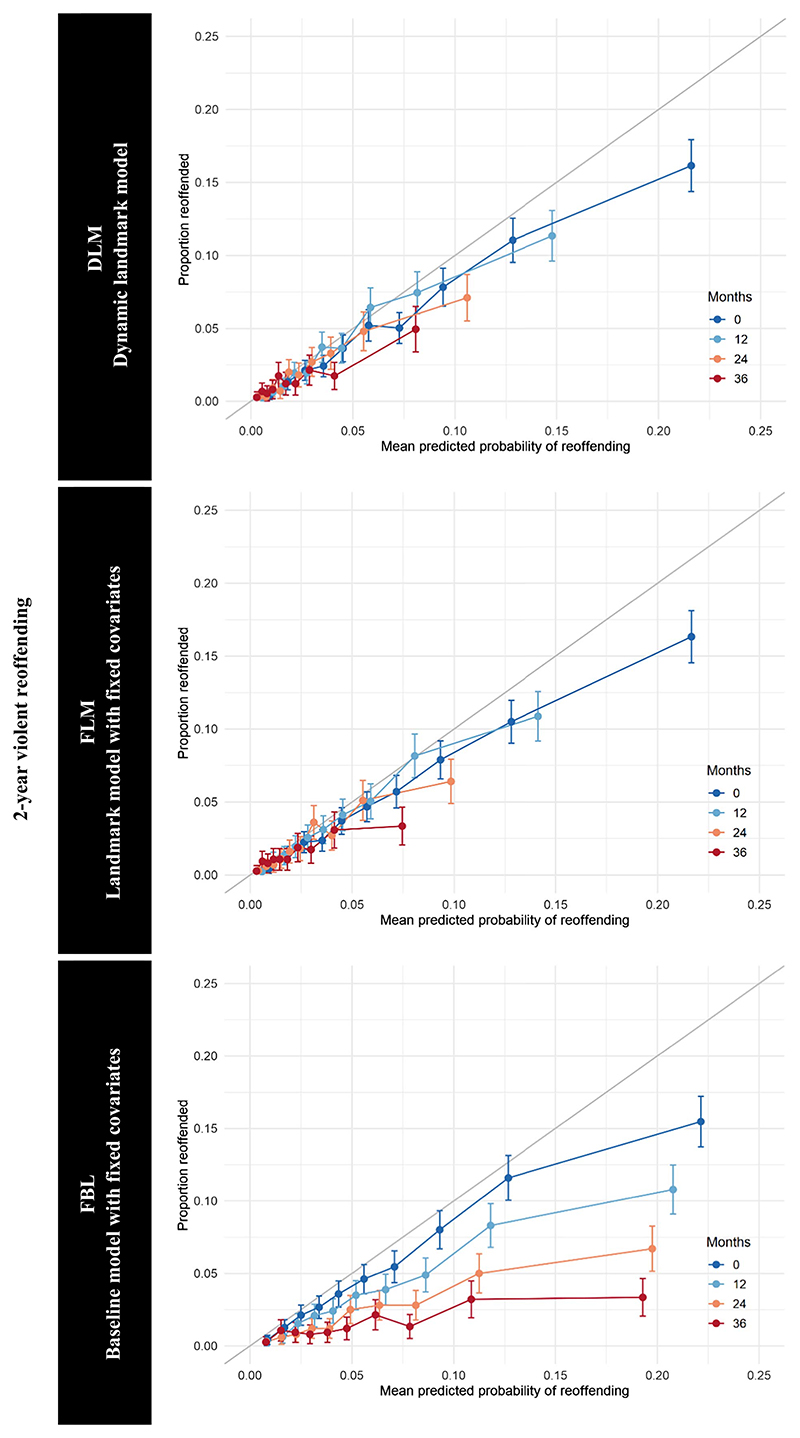
Calibration Curves for the Prediction of Violent Reoffending *Note*. Estimates for selected landmarks in the external validation data set. The individuals grouped in deciles by predicted probability of general reoffending within 2 years. See the online article for the color version of this figure.

**Figure 3:**
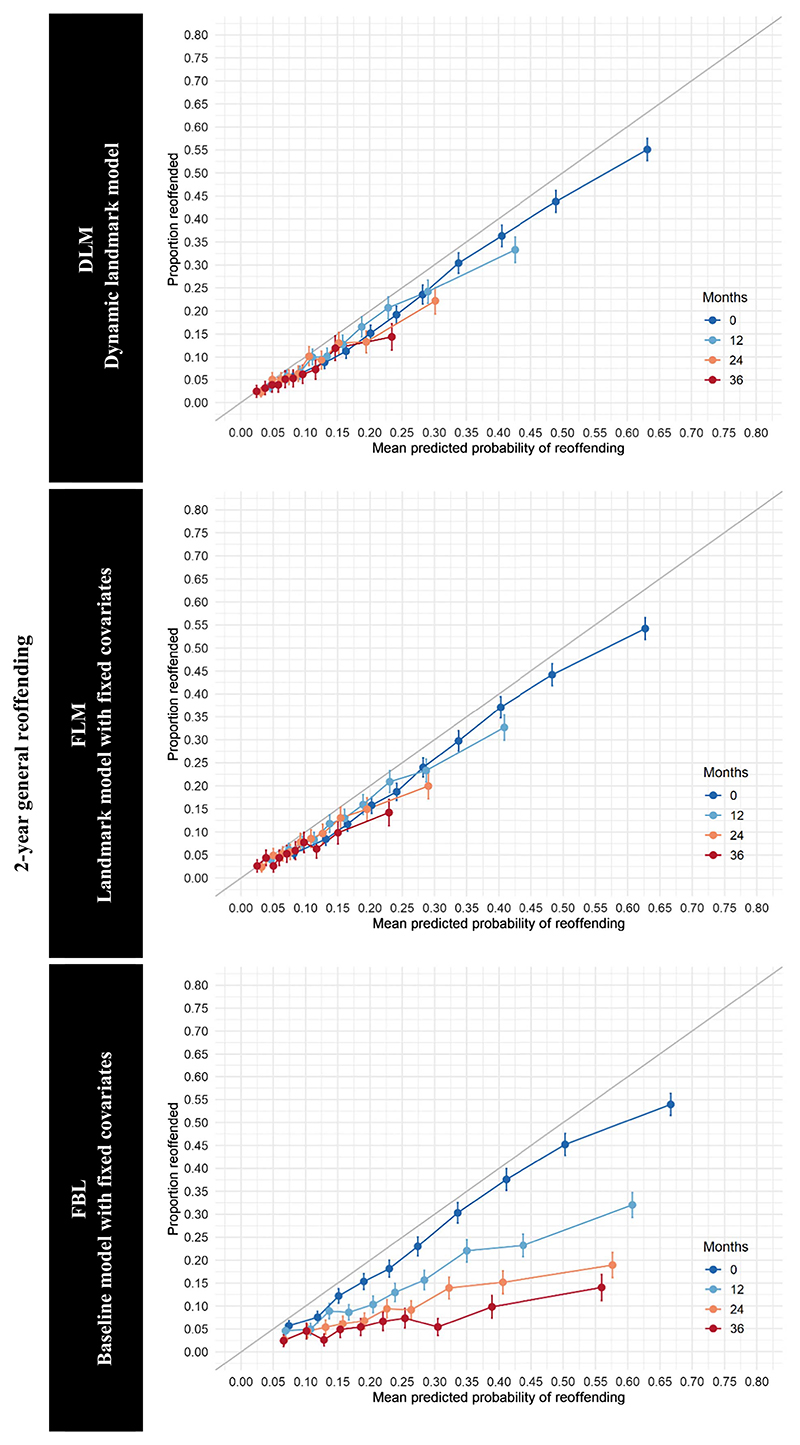
Calibration Curves for the Prediction of General Reoffending *Note*. Estimates for selected landmarks in the external validation data set. The individuals grouped in deciles by predicted probability of general reoffending within 2 years. See the online article for the color version of this figure.

DLM demonstrated good overall calibration in external validation for violent reoffending and general reoffending (Figures 2 and 3). The calibration for low- and medium-risk individuals was better than for high-risk individuals. FLM also demonstrated good calibration, and calibration curves for DLM and FLM were similar. The calibration of FBL was poor. Although the model demonstrated good calibration at baseline, over time, FLM substantially overestimated the probabilities of violent and general reoffending for all risk deciles.

Overall, DLM demonstrated very similar discrimination performance to FLM and FBL at baseline time. DLM and FLM also had similar calibration and prediction error estimates. However, both landmark models (DLM and FLM) that accounted for the offense-free time had substantially better calibration over time compared with the baseline model with fixed covariates (FLM) that did not account for offense-free time. DLM, which additionally included triggers for violence as covariates, consistently outperformed FLM over the whole follow-up period.

### Model Deployment

The final model (DLM) was translated into an online risk assessment tool, OxMore (https://oxrisk.com/oxmore/). Refer to Appendix J for the illustration of the risk assessment instrument application using hypothetical vignettes.

## Discussion

We examined the effects of modeling offense-free time within criminal recidivism risk assessment tools used for monitoring. We demonstrated that using tools for monitoring not developed for this purpose can lead to time-dependent bias that will lead to overestimation of risk over time. We have additionally demonstrated that acute adverse events (self-harm episodes, being a victim of violent crime, incident TBI, and others) contain potentially useful information about the current level of risk, that is, have predictive validity. To account for these findings, we have developed a dynamic prediction model for monitoring criminal recidivism risk over 2 years using a population sample of 43,192 individuals who received community sentences in Sweden. The model was subsequently externally validated using a sample of 16,484 individuals. The rate of violent reoffending was 7% and of general reoffending was 30% during the median follow up of 23 months. Our final model demonstrated good calibration (as indicated by calibration plots and Brier scores) and discrimination performance (c-index = 0.74 for violent reoffending, c-index = 0.69 for general reoffending).

To facilitate practical interpretation of the final model, we have additionally presented classification measures, including sensitivity and specificity, using different threshold scores. Depending on the context, these thresholds could be used to support decision making. However, careful evaluation is necessary before deciding which threshold to use in order to assess the impact of false positives or false negatives in different settings. If being classified as high risk has nonharmful implications for the community sentenced individual, such as additional psychosocial support or treatment, tolerating false positives (lower specificities) may be acceptable. However, policy-makers typically have little tolerance for false negatives (i.e., missing people who go on to commit crimes), so higher sensitivities are usually expected. If the goal is to reduce false positives and minimize the associated potential ethical, legal, and economic costs, a higher threshold may be considered. Conversely, if the aim of the criminal justice system is to introduce interventions to prevent recidivism, a lower threshold will be more appropriate. For both violent and any reoffending, our models achieved high sensitivity (>80%) and specificity (>80%), depending on the chosen thresholds. This enables the identification of high-risk individuals and effective screening out of low-risk offenders.

The final model takes into account incident adverse events during community supervision (referred to as “triggers”), changes in the supervised individual’s circumstances, and natural desistance from offending over time. The model’s performance is not worse than validated recidivism risk assessment tools (Fazel et al., 2022), which typically show performance ranging from 0.57 to 0.75 in external validation studies. However, these risk assessment tools assume the same baseline risk of reoffending over time, provided that the individual’s characteristics and environment remain unchanged. Studies on desistance from criminal behavior have demonstrated that the longer an individual remains free from offenses, the lower their likelihood of reoffending (Bushway et al., 2011; Hanson, 2018). In our study, including these periods of nonoffending significantly improved the model’s discrimination and calibration. Moreover, comparator models that did not incorporate this effect of desistance accumulated bias toward higher risk estimation over time. Furthermore, the inclusion of time-varying covariates also enhanced the model’s performance. The improvement was more pronounced in the later stages of the follow-up period, suggesting greater predictive validity of temporally proximal factors during later supervision phases. These findings underscore the importance of dynamic predictive models of reoffending considering the desistance effect to avoid biased scores for individuals not offending. Most standard risk assessment tools may overlook this risk reduction due to the absence of built-in desistance factors.

Overall, our findings support the predictive utility of time-updated modeling in justice settings, which aligns with recent evidence from other domains (e.g., mental health, cardiovascular risk) where dynamic risk factors improve forecasting accuracy (Lee et al., 2024). By incorporating real-time changes in risk estimates and modeling their decay, our study addresses a key gap in forensic risk assessment: namely, how to monitor individuals whose risk profiles evolve over time. This novel feature has important implications for tailoring supervision and interventions more responsively. As we have shown, failure to account for risk decay may result in the accumulation of miscalibration over the course of an individual’s community supervision and in overestimated risk scores. There are risk-based tools that recommend repeated use, for instance, every 6 months in the case of the Historical Clinical Risk Management-20 Version 3 (Douglas et al., 2014) and the Short-Term Assessment of Risk and Treatability (Webster et al., 2006). However, while these tools allow for reassessment, they do not produce risk estimates that account for the effects of risk decay. As a result, they can generate miscalibrated (and hence inaccurate) predictions that do not reflect the individual’s actual level of risk.

The dynamic structure of the current model makes it suitable for ongoing risk monitoring during community supervision. While we did not undertake direct comparisons with existing instruments, our findings suggest that explicitly modeling acute risk factors and offence-free time can yield potential improvements in predictive performance. This approach can be complementary to current risk assessment instruments. In contrast to existing instruments, which are typically administered either at the beginning of a community sentence or at periodic intervals, this model could allow supervision services to update risk assessments by taking offence-free time into account. It also enables the modeling of acute changes in risk following adverse events such as psychiatric hospitalization, drug or alcohol overdose, being a victim of a violent crime, or a self-harm episode. Because it relies on routinely collected administrative data, the model can be integrated into existing or new electronic case management systems. This facilitates a more responsive approach to supervision, where meaningful changes in an individual’s circumstances can trigger timely interventions, such as increased monitoring, clinical reassessment, or referral to support services. The model further incorporates new risk factors such as head injury, which may be important therapeutic targets, and it captures risk dynamically, aligning with clinical understanding of risk as a changing process.

By relying on routinely collected administrative data, the model can be integrated into existing digital case management systems with minimal disruption, allowing for risk alerts or integration with dashboards for probation officers. This facilitates a more responsive approach to supervision, where meaningful changes in an individual’s circumstances can trigger timely interventions, such as increased monitoring, clinical reassessment, or referrals to support services. We have also translated the model into a scalable, web-based, and transparently reported tool (OxMore), which allows for implementation in settings with limited resources, including low- and middle-income countries. Thus, the present tool has value as a screening and monitoring aid that can be implemented at a population level using routinely collected data, to flag higher risk cases and track changes over time, especially where resources or specialized training for comprehensive assessments are not available.

One reported example of an actuarial risk assessment tool that incorporates offense-free time in dynamic violence risk assessment in individuals under community sentences is the Offender Assessment System (OASys) used in the United Kingdom (Howard & Dixon, 2012). OASys involves a comprehensive suite of tools, requiring significant time investment for completion. Due to its complexity, it may not be suitable as a screening instrument and adapting it for use in other countries could pose substantial challenges. In comparison, the present model is scalable, transparently reported, and incorporates acute risk triggers over time. This type of dynamic risk assessment can provide a more personalized and responsive approach to informing supervision and resource allocation. Some structured professional judgment instruments direct assessors to consider triggers and warning signs when formulating risk at the individual level. This model differs by testing the predictive utility of such triggers statistically in a large population-based data set. We deployed the final model as an online tool for assessing criminal recidivism risk called OxMore (see https://oxrisk.com/oxmore/). This tool includes 23 predictors for violent reoffending and 28 predictors for general reoffending. It can be used to assess changes in the risk of general and violent reoffending during the 3 years following the start of a community sentence. It can be viewed as a scalable screening and monitoring aid, designed to flag cases at elevated risk using routinely collected register data or as part of routine systems. Its dynamic structure allows risk estimates to be updated over time, thereby capturing meaningful changes in circumstances (e.g., offense-free periods, new adverse events) that may not be reflected in static or baseline-only assessments. In this way, OxMore can support resource allocation and supervision planning, complementing existing approaches within a tiered risk assessment pipeline.

### Strengths and Limitations

The study has several strengths. We developed a risk assessment tool using data from a large cohort of individuals who received community sentences. Drawing on a large population cohort ensured the stability of the derived models and removed selection biases. The model also included predictors that allow for generalizability across different countries and legal contexts. This adaptability facilitates straightforward recalibration and the wider deployment of the developed dynamic risk assessment tool beyond Sweden. Furthermore, we provided a comprehensive account of the calibration process, which is a strength given it is frequently missed (Van Calster et al., 2019). The developed risk assessment tool demonstrates good discrimination and a low level of optimism, suggesting robust performance beyond the derivation sample. Another strength is that the developed tool takes into consideration desistance from crime, leading to more precise reoffending risk estimates over time in comparison to other commonly employed models. The tool generates probabilities of reoffending and baseline risk for a given offense-free period. Given the primary aim was to develop a predictive tool, we prioritized maximizing predictive validity over explanatory modeling. As such, interaction terms were not included unless they provided clear evidence of improving predictive performance in exploratory analyses. This approach was intended to balance parsimony and interpretability with the practical goal of building a tool that generalizes well to new data.

The study also has several limitations. First, population registers lack data regarding potentially significant predictors of criminal recidivism identified in published research, such as the absence of social support and association with antisocial peers. Moreover, we lacked information about intervention programs that sentenced individuals might have undergone. The successful completion of a rehabilitation program could potentially constitute a strong desistance factor. Furthermore, sociodemographic covariates are only recorded in population registers once annually for all individuals in Sweden. This cross-sectional methodology limits the validity of sociodemographic variables as dynamic predictors, as they are unlikely to capture abrupt changes in an individual’s life circumstances, such as the loss of employment.

Second, the examined predictors were also restricted to variables available in administrative registers. In case of data from medical registers on psychiatric diagnoses and triggers, this approach could potentially bias estimates of their effect due to the likelihood of underreporting. This bias is more likely to affect the negative predictive value of the developed models. A lower negative predictive value means the model may incorrectly classify some high-risk individuals as low risk. The outcome measure was based on officially recorded conviction data, which will underestimate the true reoffending prevalence by omitting unreported or unprocessed offences. However, outcomes used were formally investigated and recorded, which suggests that tools trained on these data may demonstrate higher specificity in identifying recidivism risk (and likely capture more serious offences with more societal costs).

Other predictors examined were also restricted to variables available in administrative registers. Although valid and reliable, they are limited in breadth or depth. Constructs such as psychopathy, impulsivity/dysregulation, antisocial peers, and noncompliance, which were shown to be predictive of criminal recidivism (Lovatt et al., 2025; Moosburner et al., 2024), are not routinely available in such data. Similarly, register-based indicators such as hospitalization or presence of a mental disorder can flag elevated risk but may provide less clinically actionable detail than symptom-level data. Thus, such novel tools can be embedded within a tiered risk assessment pipeline, where initial screening and monitoring can be complemented by more comprehensive assessment, which can provide a framework for risk formulation and intervention planning.

Third, the current tool was developed and validated using Swedish register data and its statistical estimates may not generalize to other countries. Differences in health care systems, treatment provision, and justice processes are likely to influence recidivism rates. Replication would require access to comparable register systems, which may be feasible in some countries or large research cohorts. As with all prediction models, recalibration and external validation should be integrated into implementation plans in new settings.

## Conclusion

Using data from a large population-based cohort of individuals given community sentences, we developed an evidence-based, scalable, and flexible tool for dynamic risk assessment of general and violent reoffending in community sentenced individuals. The tool demonstrated good predictive performance (based on discrimination and calibration) for both outcomes and could be considered for risk monitoring during postsentence supervision. Additionally, we investigated the impact of using criminal recidivism risk prediction models without accounting for offense-free time and showed that they accumulate significant inaccuracies over time and should not be employed without appropriate recalibration.

## Supplementary Material

Appendix

## Figures and Tables

**Table 1:** Baseline Characteristics and Follow-Up Data of Adults Receiving Community Sentences in Sweden

Variable	Derivation sample	Validation sample	Total cohort
Number of individuals	43,192 (100.0%)	16,484 (100.0%)	59,676 (100.0%)
Follow-up data			
Number of person-years at risk	2,240.4	852.7	3,093.1
Incidents of general reoffending	13,300 (30.8%)	5,007 (30.4%)	18,307 (30.7%)
Incidents of violent reoffending	3,220 (7.5%)	1,196 (7.3%)	4,416 (7.4%)
Imprisoned during follow up	2,489 (5.8%)	968 (5.9%)	3,457 (5.8%)
Emigrated during follow up	549 (1.3%)	237 (1.4%)	786 (1.3%)
Died during follow up	1,337 (3.1%)	542 (3.3%)	1,879 (3.1%)
Covariates at baseline			
Sex (male)	36,794 (85.2%)	14,021 (85.1%)	50,815 (85.2%)
Median age	32 (IQR: 23–45)	31 (IQR: 22–45)	32 (IQR: 23–45)
Prior criminal history	30,050 (69.6%)	11,376 (69.0%)	41,426 (69.4%)
Prior conviction for a violent crime	13,629 (31.6%)	5,124 (31.1%)	18,753 (31.4%)
Prior prison sentence	7,781 (18.0%)	2,975 (18.0%)	10,756 (18.0%)
Violent index offense	20,513 (47.5%)	7,732 (46.9%)	28,245 (47.3%)
Married or in a registered partnership	6,505 (15.1%)	2,284 (13.9%)	8,789 (14.7%)
Employed	20,354 (47.1%)	7,133 (43.3%)	27,487 (46.1%)
Recipient of income support	12,037 (27.9%)	5,275 (32.0%)	17,312 (29.0%)
Unstable housing situation	544 (1.3%)	254 (1.5%)	798 (1.3%)
Highest level of education			
<9 years	2,187 (5.1%)	819 (5.0%)	3,006 (5.0%)
9–11 year	35,381 (81.9%)	13,645 (82.8%)	49,026 (82.2%)
≥12 years	4,223 (9.8%)	1,527 (9.3%)	5,750 (9.6%)
History of self-harm	3,934 (9.1%)	1,577 (9.6%)	5,511 (9.2%)
Previous psychiatric disorders at baseline			
Any psychiatric disorder	19,454 (45.0%)	7,425 (45.0%)	26,879 (45.0%)
Any psychiatric disorder (other than substance use)	13,731 (31.8%)	5,582 (33.9%)	19,313 (32.4%)
Any severe psychiatric disorder	2,250 (5.2%)	784 (4.8%)	3,034 (5.1%)
Schizophrenia spectrum disorder	1,463 (3.4%)	546 (3.3%)	2,009 (3.4%)
Bipolar disorder	787 (1.8%)	238 (1.4%)	1,025 (1.7%)
Substance (drug or alcohol) use disorder	13,591 (31.5%)	4,955 (30.1%)	18,546 (31.1%)
Alcohol use disorder	9,507 (22.0%)	3,200 (19.4%)	12,707 (21.3%)
Drug use disorder	7,702 (17.8%)	3,047 (18.5%)	10,749 (18.0%)

*Note*. The follow up and censoring data are presented for violent reoffending outcome. IQR = interquartile range.

## Public Significance Statement

This tool offers a new approach to risk assessment for community-sentenced individuals, helping improve monitoring during postsentence supervision and potentially reducing reoffending rates. Our findings also underscore the importance of recalibrating risk tools over long follow-up times, as inaccuracies can develop if offense-free periods are not considered. Such advancements in risk assessment could lead to more reliable, fair, and adaptable practices in community sentence supervision.
