# Supplementary material for: Dynamic Prediction of Reoffending in Individuals Given Community Sentences: Development and Validation of a Novel Risk Monitoring Assessment Tool (OxMore)
Source: Law Hum Behav. Author manuscript; Available in PMC 2026 Apr 1. (PMC7618717; doi:10.1037/lhb0000641)
Supplement: Appendix [file EMS212265-supplement-Appendix.pdf]

Appendix A

Predictive Model Development Protocol

Section/topic	Item	Stage	Checklist item	Page
			Title and abstract	
Title	1	D;V	Identify the study as developing and/or validating a multivariable prediction model, the target population, and the outcome to be predicted.	1
Abstract	2	D;V	Provide a summary of objectives, study design, setting, participants, sample size, predictors, outcome, statistical analysis, results, and conclusions.	1
			Introduction	
Background and objectives	3a	D;V	Explain the medical context (including whether diagnostic or prognostic) and rationale for developing or validating the multivariable prediction model, including references to existing models.	5–6
	3b	D;V	Specify the objectives, including whether the study describes the development or validation of the model or both.	5–6
			Methods	
Source of data	4a	D;V	Describe the study design or source of data (e.g., randomized trial, cohort, or registry data), separately for the development and validation data sets, if applicable.	6
	4b	D;V	Specify the key study dates, including start of accrual; end of accrual; and, if applicable, end of follow up.	7
Participants	5a	D;V	Specify key elements of the study setting (e.g., primary care, secondary care, general population) including number and location of centres.	7
	5b	D;V	Describe eligibility criteria for participants.	7
	5c	D;V	Give details of treatments received, if relevant.	N/A
Outcome	6a	D;V	Clearly define the outcome that is predicted by the prediction model, including how and when assessed.	8
	6b	D;V	Report any actions to blind assessment of the outcome to be predicted.	N/A
Predictors	7a	D;V	Clearly define all predictors used in developing or validating the multivariable prediction model, including how and when they are measured.	8–9, Appendix B
	7b	D;V	Report any actions to blind assessment of predictors for the outcome and other predictors.	N/A
Sample size	8	D;V	Explain how the study size is arrived at.	7, Appendix B
Missing data	9	D;V	Describe how missing data are handled (e.g., complete-case analysis, single imputation, multiple imputation) with details of any imputation method.	10
Statistical analysis methods	10a	D	Describe how predictors are handled in the analyses.	11–13, Appendix B
	10b	D	Specify type of model, all model-building procedures (including any predictor selection), and method for internal validation.	11–13
	10c	V	For validation, describe how the predictions are calculated.	12–13, Appendix B
	10d	D;V	Specify all measures used to assess model performance and, if relevant, to compare multiple models.	13–14
	10e	V	Describe any model updating (e.g., recalibration) arising from the validation, if done.	13–14

(*Appendices continue*)

Appendix A (*continued*)

Section/topic	Item	Stage	Checklist item	Page
Risk groups	11	D;V	Provide details on how risk groups are created, if done.	N/A
Development versus validation	12	V	For validation, identify any differences from the development data in setting, eligibility criteria, outcome, and predictors.	Table 1, Appendix B
			Results	
Participants	13a	D;V	Describe the flow of participants through the study, including the number of participants with and without the outcome and, if applicable, a summary of the follow-up time. A diagram may be helpful.	Table 1
	13b	D;V	Describe the characteristics of the participants (basic demographics, clinical features, available predictors), including the number of participants with missing data for predictors and outcome.	Table 1, Appendix C
	13c	V	For validation, show a comparison with the development data of the distribution of important variables (demographics, predictors and outcome).	Table 1
Model development	14a	D	Specify the number of participants and outcome events in each analysis.	Table 1
	14b	D	If done, report the unadjusted association between each candidate predictor and outcome.	N/A
Model specification	15a	D	Present the full prediction model to allow predictions for individuals (i.e., all regression coefficients, and model intercept or baseline survival at a given time point).	Appendix F
	15b	D	Explain how to the use the prediction model.	Appendix F
Model performance	16	D;V	Report performance measures (with CIs) for the prediction model.	17, Figures 1–3, Appendix G
Model-updating	17	V	If done, report the results from any model updating (i.e., model specification, model performance).	Appendices D–F
			Discussion	
Limitations	18	D;V	Discuss any limitations of the study (such as nonrepresentative sample, few events per predictor, missing data).	25–26
Interpretation	19a	V	For validation, discuss the results with reference to performance in the development data, and any other validation data.	23–24
	19b	D;V	Give an overall interpretation of the results, considering objectives, limitations, results from similar studies, and other relevant evidence.	23–25
Implications	20	D;V	Discuss the potential clinical use of the model and implications for future research.	23–25
			Other information	
Supplementary information	21	D;V	Provide information about the availability of supplementary resources, such as study protocol, Web calculator, and data sets.	25
Funding	22	D;V	Give the source of funding and the role of the funders for the present study.	Funding

Note. Items relevant only to the development of a prediction model are denoted by D, items relating solely to a validation of a prediction model are denoted by V, and items relating to both are denoted D;V. We recommend using the TRIPOD Checklist in conjunction with the TRIPOD Explanation and Elaboration document. TRIPOD = transparent reporting of a multivariable prediction model for individual prognosis or diagnosis; N/A = not applicable; CI = confidence interval.

(*Appendices continue*)

Appendix B

Predictive Model Derivation Data

Table B1

Covariates Used in the Dynamic Risk Assessment Model, Grouped by Inclusion Timing and Type

Covariate	Type	Description
Group 1: Fixed baseline measures (included by default)		
Sex	Binary	Male/Female
Any previous offense	Binary	Any prior offense committed before the index offense
Previous violent offense	Binary	Any violent offense committed before the index offense
Violent index offense	Binary	The most recent offense is a homicide, assault, robbery, arson, any sexual offense (rape, sexual coercion, child molestation, indecent exposure, or sexual harassment), illegal threats, or intimidation.
Prior imprisonment	Binary	Any prison sentence served before the index offense
Group 2: Baseline measures dynamically updated during the follow up (included by default)		
Current age	Continuous	Age in years
Current civil status	Binary	Single/Married. Recorded once a year in November. The record done within a year prior to a given landmark will be used. If the record is missing, then its value will be imputed.
Current years of education	Categorical	<9 years, 9–11 years, ≥12 years. Recorded once a year in November. The record done within a year prior to a given landmark will be used. If the record is missing, then its value will be imputed.
Current employment	Binary	Employment status. Recorded once a year in November. The record is done within a year prior to a given landmark will be used. If the record is missing, then its value will be imputed.
Current receipt of income support	Binary	Receipt of financial support due to low income. Recorded once a year in November. The record done within a year prior to a given landmark will be used. If the record is missing, then its value will be imputed.
Current unstable housing	Binary	More than 3 changes of address during last year. Recorded once a year in November. The record done within a year prior to a given landmark will be used. If the record is missing, then its value will be imputed.
Any prior mental disorder	Binary	Any lifetime psychiatric diagnosis prior to a given landmark. *ICD*-10 codes: F00–F99.
Prior severe mental disorder	Binary	Any lifetime diagnosis of schizophrenia spectrum or bipolar disorder prior to a given landmark. *ICD*-10 codes: F20–F29, F30–F31
Prior alcohol use disorder	Binary	Any lifetime diagnosis of alcohol use disorder prior to a given landmark. *ICD*-10 codes: F10.
Prior drug use disorder	Binary	Any lifetime diagnosis of drug use disorder prior to a given landmark. *ICD*-10 codes: F11–F19.
Group 3: Fixed baseline measures (potentially included)		
Civil status at sentence	Binary	Single/Married. Recorded once a year in November. The record done within a year prior to the index sentence will be used. If the record is missing, then its value will be imputed.
Employment at sentence	Binary	Employment status. Recorded once a year in November. The record done within a year prior to the index sentence will be used. If the record is missing, then its value will be imputed.
Receipt of income support at sentence	Binary	Receipt of financial support due to low income. Recorded once a year in November. The record done within a year prior to the index sentence will be used. If the record is missing, then its value will be imputed.
Unstable housing at sentence	Binary	More than 3 changes of address during last year. Recorded once a year in November. The record done within a year prior to the index sentence will be used. If the record is missing, then its value will be imputed.
History of self-harm or suicide attempts	Binary	Any history of self-harm prior to the index sentence. *ICD*-10 codes: X60–X84, Y10–Y34.
Group 4: Events occuring during the follow-up period (potentially included)		
Being a victim of a violent assault	Binary	Data from medical records. Measured continuously.
Time since the last assault	Continuous	In weeks
Traumatic brain injury (TBI)	Binary	Measured continuously. *ICD* codes: S01, S02.0–S02.3, S02.7–S02.9, S04, S06.0–S06.9, S07.0–S07.1, S07.8–S07.9, S09.7–S09.9, T01.0, T02.0, T04.0, T06.0, T90.1–T90.2, T90.4–T90.5, T90.8–T90.9
Time since the last TBI	Continuous	In weeks
Self-harm episode	Binary	Self-harm episode or a suicide attempt during a follow up. Measured continuously. *ICD* codes: X60–X84, Y10–Y34

(*table continues*)

(*Appendices continue*)

Table B1 (*continued*)

Covariate	Type	Description
Time since the last self-harm episode	Continuous	In weeks
Injuries from other causes (not TBI, not self-harm)	Binary	Measured continuously. *ICD* codes: V00–V99, W00–W99, X00–X59
Time since the last injury	Continuous	In weeks
Any psychiatric hospitalization	Binary	Measured continuously. Inpatient only. *ICD*-10 codes: F00–F99.
Time since last psychiatric hospitalization	Continuous	In weeks
Substance intoxication	Binary	Measured continuously. *ICD* codes: F10.0–F12.0, F14.0–F16.0, F19.0
Time since the last intoxication	Continuous	In weeks

Note. TBI = traumatic brain injury; *ICD*-10 = *International Statistical Classification of Diseases*, 10th revision.

Table B2

The Rule for Splitting the Full Data Set Into Derivation and External Validation Samples

Group I: Major urban centers	Group II: Counties with major urban centers removed	Group III: Counties with small population	Group IV: Counties with medium population
1 Stockholm City North	1 Stockholm County other	7 Kronoberg	3 Uppsala
1 Stockholm City South	12 Skåne other	9 Gotland	4 Södermanland
12 Malmö	14 Västra Götaland other	10 Blekinge	5 Östergötland
14 Gothenburg		23 Jämtland	6 Jönköping
			8 Kalmar
			13 Halland
			17 Värmland
			18 Örebro
			19 Västmanland
			20 Dalarna
			21 Gävleborg
			22 Västernorrland
			24 Västerbotten
			25 Norrbotten

Note. Regions will be selected at random, with equal probability, as follows: (a) One region from Group I. (b) One region from Group II (under the constraint that no more than one region in Groups I and II from the same county can be selected). (c) One region from Group III. (d) Sequentially select from Group IV until the number of individuals in the external validation sample is equal to or exceeds 20% of the entire data set.

Appendix C

The Rates of Missing Data for Sociodemographic Covariates by the Time After Being Sentenced

Months after sentence	*N*	Employment	Marital status	Education level	Receipt of income support	House changes
Derivation sample						
0	43,192	0.8%	0.8%	3.2%	0.8%	1.0%
12	33,868	0.9%	0.9%	2.0%	0.9%	1.0%
24	26,395	0.5%	0.5%	1.5%	0.5%	0.5%
36	19,750	0.4%	0.4%	1.2%	0.4%	0.5%
External validation sample						
0	16,484	0.5%	0.5%	3.0%	0.5%	0.6%
12	12,884	0.7%	0.7%	1.9%	0.7%	0.7%
24	9,992	0.4%	0.4%	1.4%	0.4%	0.4%
36	7,472	0.2%	0.2%	1.3%	0.2%	0.3%

(*Appendices continue*)

Appendix D

Stepwise Variable Selection Results for 2-Year Violent and General Reoffending Models (First Iteration)

	2-year violent reoffending			2-year general reoffending		
Covariate	Included?	Eliminated at step …	p value at elimination	Included?	Eliminated at step …	p value at elimination
**Triggers**						
Being a victim of a violent assault	✓			✓		
Prior week	✗	4	.788	✓		
Prior month	✓			✗	3	.626
Traumatic brain injury (TBI)	✗	9	.489	✗	2	.908
Prior week	✗	2	.767	✗	7	.371
Prior month	✗	3	.861	✗	8	.514
Self-harm episode	✗	10	.480	✗	10	.174
Prior week	✗	6	.709	✗	5	.471
Prior month	✗	8	.547	✗	1	.915
Injuries from other causes	✓			✓		
Prior week	✓			✓		
Prior month	✗	7	.580	✓		
Any psychiatric hospitalization	✓			✓		
Prior week	✗	5	.691	✓		
Prior month	✗	1	.833	✗	6	.412
Substance intoxication	✗	12	.393	✓		
Prior week	✗	15	.212	✗	4	.561
Prior month	✓			✓		
**Demographics at baseline**						
Civil status (single)	✗	13	.217	✓		
Employed	✓			✓		
Receipt of income support	✗	14	.217	✓		
Unstable housing	✗	16	.211	✗	9	.182
**Mental health at baseline**						
History of self-harm or suicide attempts	✗	11	.434	✓		

Note. Variable selection results in the subset of candidate variables (covariates of Groups 3 and 4 in the protocol). The model is trained on 10 imputations. The estimates and their standard errors are combined using Rubin's rule. The selection is performed by backward elimination with the exclusion threshold of $p = .157$. Variables included in the model by default are not subjected to backward elimination.

(*Appendices continue*)

Appendix E

Variable Selection Results (Second Iteration)

Table E1

Stepwise Variable Selection Results for 2-Year Violent and General Reoffending Models (Second Iteration)

Covariate	2-year violent reoffending			2-year general reoffending		
	Included?	Eliminated at step …	*p* value at elimination	Included?	Eliminated at step …	*p* value at elimination
Triggers						
Being a victim of a violent assault	✓			✓		
Prior week	✗	1	.840	✓		
Prior month	✓			✗	1	.608
TBI or injuries from other causes	✓			✓		
Prior week	✓			✓		
Prior month	✗	2	.792	✓		
Any psychiatric hospitalization	✓			✓		
Prior week	✗	4	.850	✓		
Prior month	✗	3	.694	✗	3	.338
Substance intoxication	✗	6	.398	✓		
Prior week	✗	9	.197	✗	2	.551
Prior month	✓			✓		
Mental health (current)						
Any prior self-harm or suicide attempt	✗	5	.603	✓		
Demographics at baseline						
Employed	✓			✓		
Receipt of income support	✗	7	.213	✓		
Unstable housing	✗	8	.212	✗	4	.176

Note. Variable results for the subset of reoperationalized candidate variables. The variables included covariates of Groups 3 and 4 with the following changes: (a) "TBI" and "Injuries from other causes" are combined into one variable "TBI or injuries from other causes"; (b) "Self-harm during the follow-up" and "History of self-harm of suicide attempts" are combined into "Any prior self-harm or suicide attempt"; (c) "Marital status at sentence variable was eliminated." The model was trained on 10 imputations. The estimates and their standard errors are combined using Rubin's rule. The selection was performed by backward elimination with the exclusion threshold of $p = .157$. The subset of variables included in the mode by default: sex (male), current age, current civil status, current years of education, current employment, current receipt of income support, current unstable housing, any prior mental disorder, prior severe mental disorder, prior alcohol use disorder, prior drug use disorder, any previous offense, previous violent offense, violent index offense, and prior imprisonment. Variables included in the model by default are not subjected to backward elimination. TBI = traumatic brain injury.

Table E2

Internal Validation Results Using Harrell's Bias Correction for 2-Year Violent and General Reoffending Models

Metric	Violent reoffending	General reoffending
Apparent c-index (%)	74.60	69.49
Bootstrapped c-index (%)		
Fit on a bootstrapped sample	74.68 (73.83–75.50)	69.51 (69.06–69.97)
Fit on the original sample	74.51 (74.39–74.60)	69.45 (69.41–69.49)
Optimism-corrected c-index (%)	74.44	69.43

Note. Ten imputed data sets, 200 bootstrap iterations for each imputed data sets (2,000 bootstrap iteration in total). The 95% confidence intervals are estimated from quantiles of the bootstrapped values. Values are in parentheses 95% confidence interval.

(*Appendices continue*)

Appendix F

Formula

The model estimates the probability of an individual to commit a new offense or a new violent offense in the next 2 years after the time of an assessment. The assessment can be conducted at any point within 3 years after an individual received a community sentence. The probability of reoffending is calculated using the formula:

$$P(\text{reoffending}) = 1 - \exp\left(-\mathrm{BH}_{\mathrm{LM}}\right)^{\exp\left(\sum \beta \times \mathrm{RF}\right)}, \quad \text{(F1)}$$

where $\mathrm{BH}_{\mathrm{LM}}$ is the baseline hazard corresponding to a given landmark (in months since sentence), beta representing coefficients for the corresponding outcome, and RF is a presence or an absence of a given risk factor (coded as 1 or 0, respectively). The list of risk factors and corresponding coefficients are in the table below. The relevant baseline hazard estimations are provided in the plots below the table. General and violent reoffending are estimated using the same formula, but with different coefficients and baseline hazards. Note that some variables are only used for general offending predictions.

Baseline hazards estimates correspond to the time of the assessment. To estimate the risk at the time of receiving a community sentence, use baseline hazard corresponding to landmark 0. To estimate the risk at 6 months after receiving a community sentence, use landmark 6.

Table F1
Beta Coefficients for 2-Year Violent and General Reoffending: Full Model Stratified by Covariate Block

Variable	Coefficient (β) 2-year violent reoffending	Coefficient (β) 2-year general reoffending
Block 1. Current status		
This block includes		
(a) Items that denote the current status (i.e., current employment, housing, etc.)		
(b) Items that code past events that might have happened at any point before the current assessment (i.e., receiving a psychiatric diagnosis)		
1.1 Demographics		
Sex (male)	0.618	0.408
Age (in years)	−0.034	−0.025
Civil status (single)	−0.135	−0.059
Education level:		
9–11 years of education (finished high school)	−0.197	−0.107
12 or more years of education (undergraduate and further)	−0.514	−0.354
Currently employed	−0.215	−0.213
Unstable housing situation	0.080	0.024
Receipt of income support	0.311	0.187
1.2 Mental health history		
History of any psychiatric disorder (excluding drug or alcohol use)	0.178	−0.023
History of severe psychiatric disorder (schizophrenia spectrum or bipolar disorder)	0.140	−0.070
History of alcohol use disorder	0.249	0.066
History of drug use disorder	0.072	0.393
History of self-harm or suicide attempts	NA	−0.055
1.3 Criminal history		
Any prior offense (excluding the index offense)	0.368	0.565
Any prior violent offense (excluding the index offense)	0.602	0.168
Index violent offense	0.579	−0.129
Prior imprisonment	0.380	0.349
Block 2. Status at sentence		
This block includes items that code an individual's status at the time of receiving a community sentence.		
Employed at the time of a sentence	−0.177	−0.211
Receipt of income support at the time of a sentence	NA	0.142
Block 3. Triggers and stressful events		
This block includes items that code adverse events, which might have occurred since the time of receiving a community sentence and before the current assessment. These events could be associated with an ongoing distress and disadaptation.		

(*table continues*)

(*Appendices continue*)

Table F1 (*continued*)

Variable	Coefficient (β)	
	2-year violent reoffending	2-year general reoffending
3.1 Occurred at any point since receiving a sentence		
Psychiatric hospitalization	0.326	0.100
Sustaining an injury from any cause (including traumatic brain injury, excluding self-harm)	0.227	0.172
Being a victim of a violent assault	0.301	0.254
Substance intoxication	NA	0.131
3.2 Occurred within last 30 days		
Sustaining an injury from any cause (including traumatic brain injury, excluding self-harm)	NA	0.049
Substance intoxication	0.134	0.086
Being a victim of a violent assault	0.151	0.087
3.3 Occurred within last 7 days		
Psychiatric hospitalization	NA	0.094
Sustaining an injury from any cause (including traumatic brain injury, excluding self-harm)	−0.180	−0.078

Note. NA = not applicable.

(*Appendices continue*)

Figure F1

Baseline Hazard Estimates for 2-Year General and Violent Reoffending Across Monthly Landmarks (BH_{LM})

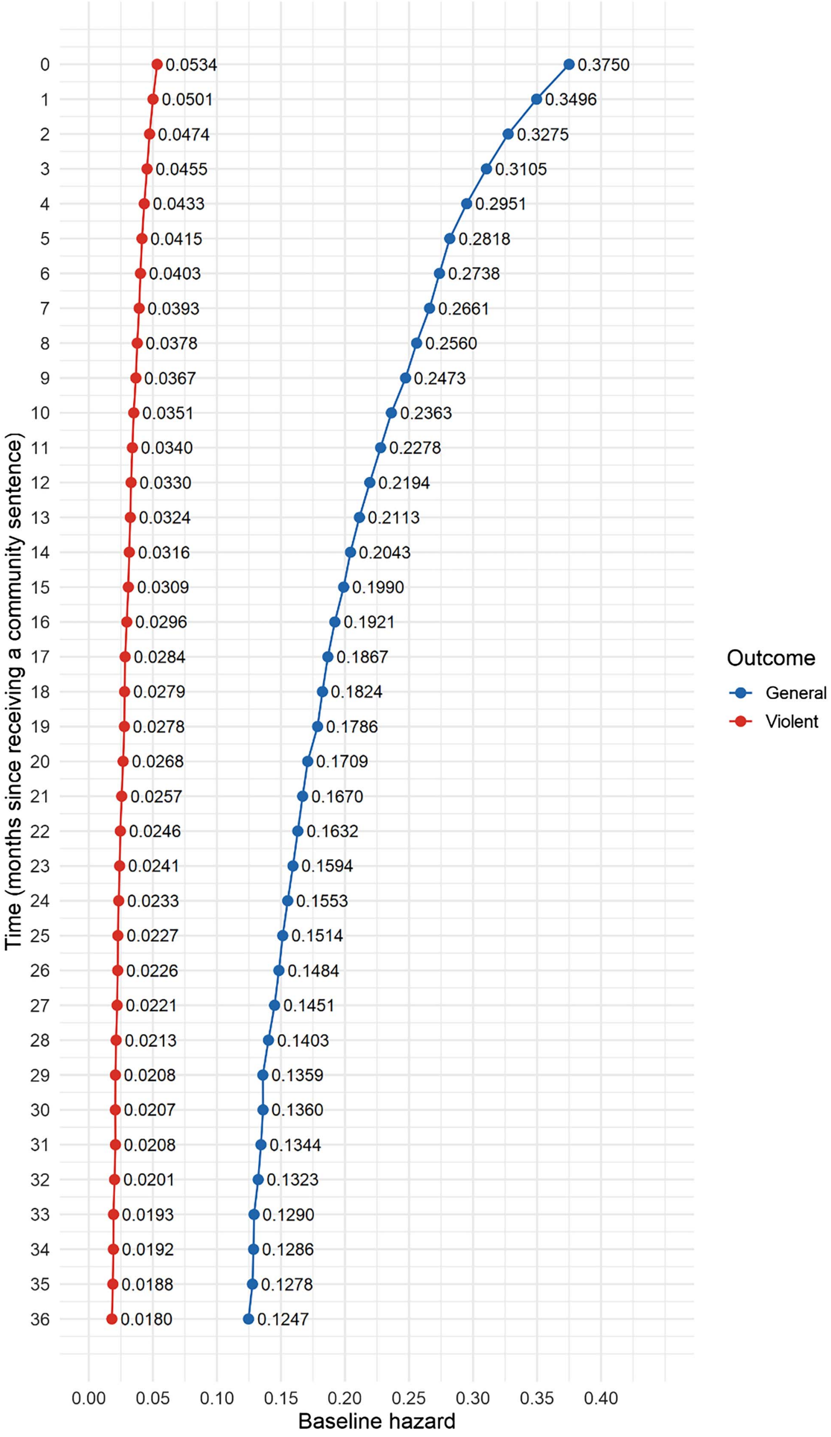

Note. See the online article for the color version of this figure.

(*Appendices continue*)

Appendix G

Cumulative Prevalence of Trigger Events Among At-Risk Individuals in Derivation and External Validation Samples Over Time

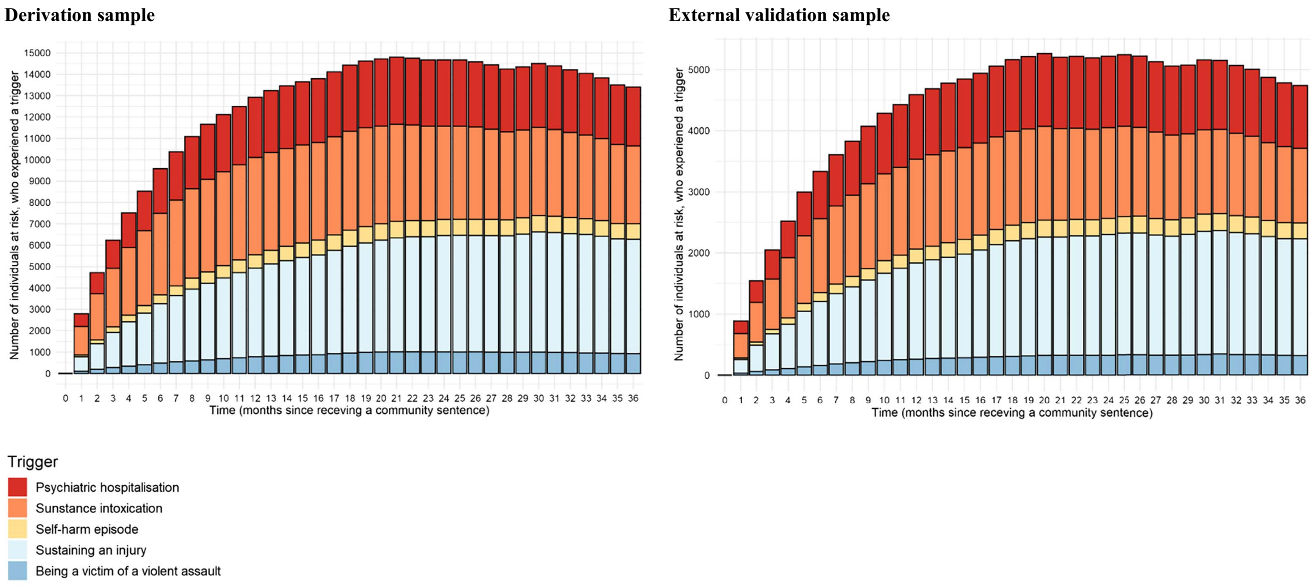

Note. See the online article for the color version of this figure.

(*Appendices continue*)

Appendix H

Violent and General Reoffending Rates in Derivation and External Validation Data Sets

Figure H1

Violent and General Reoffending Rates (as Kaplan–Meier Curves) by Time and Landmark Point in the Derivation Data Set

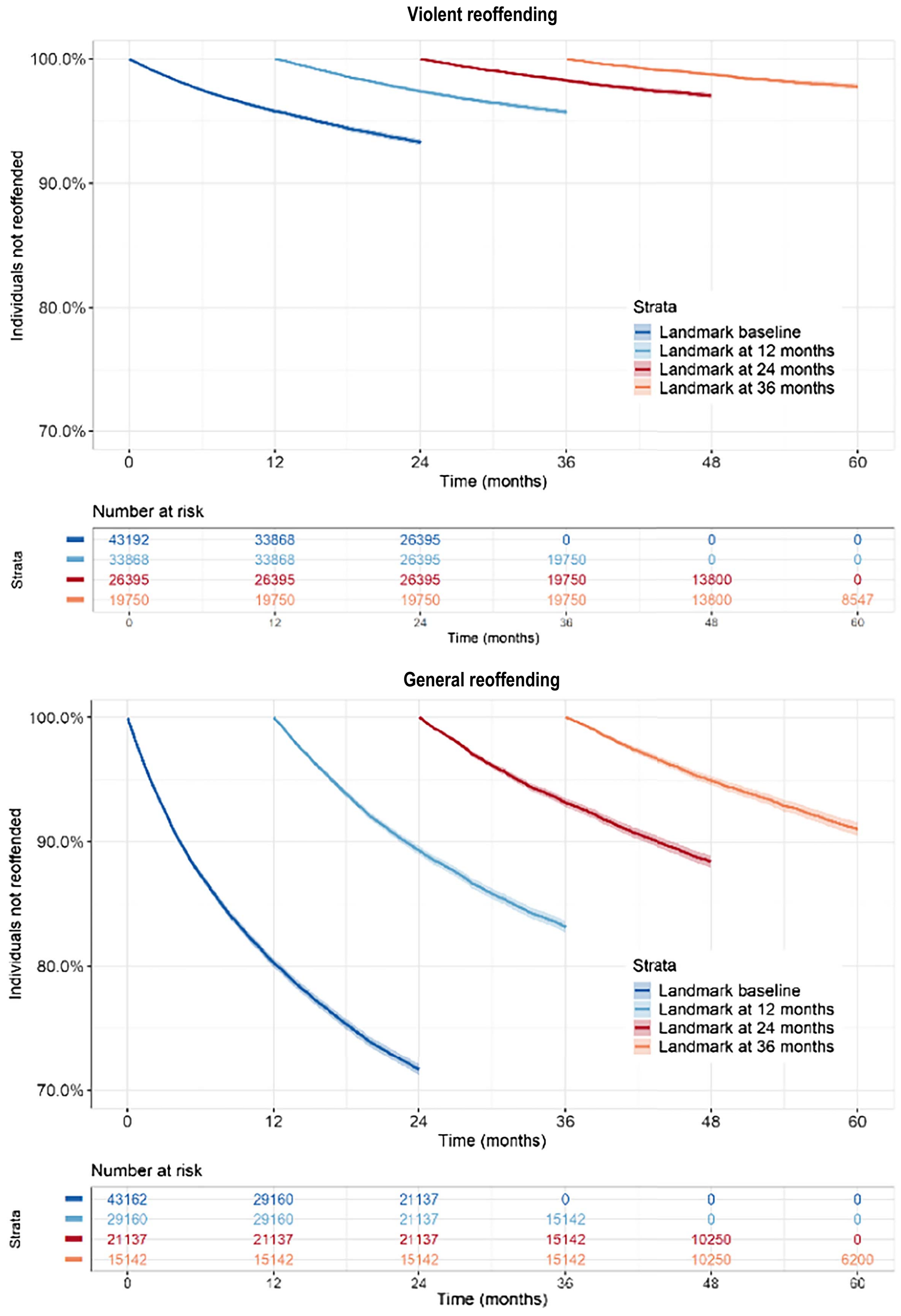

Note. See the online article for the color version of this figure.

(*Appendices continue*)

Figure H2

Violent and General Reoffending Rates (as Kaplan–Meier Curves) by Time and Landmark Point in the External Validation Data Set

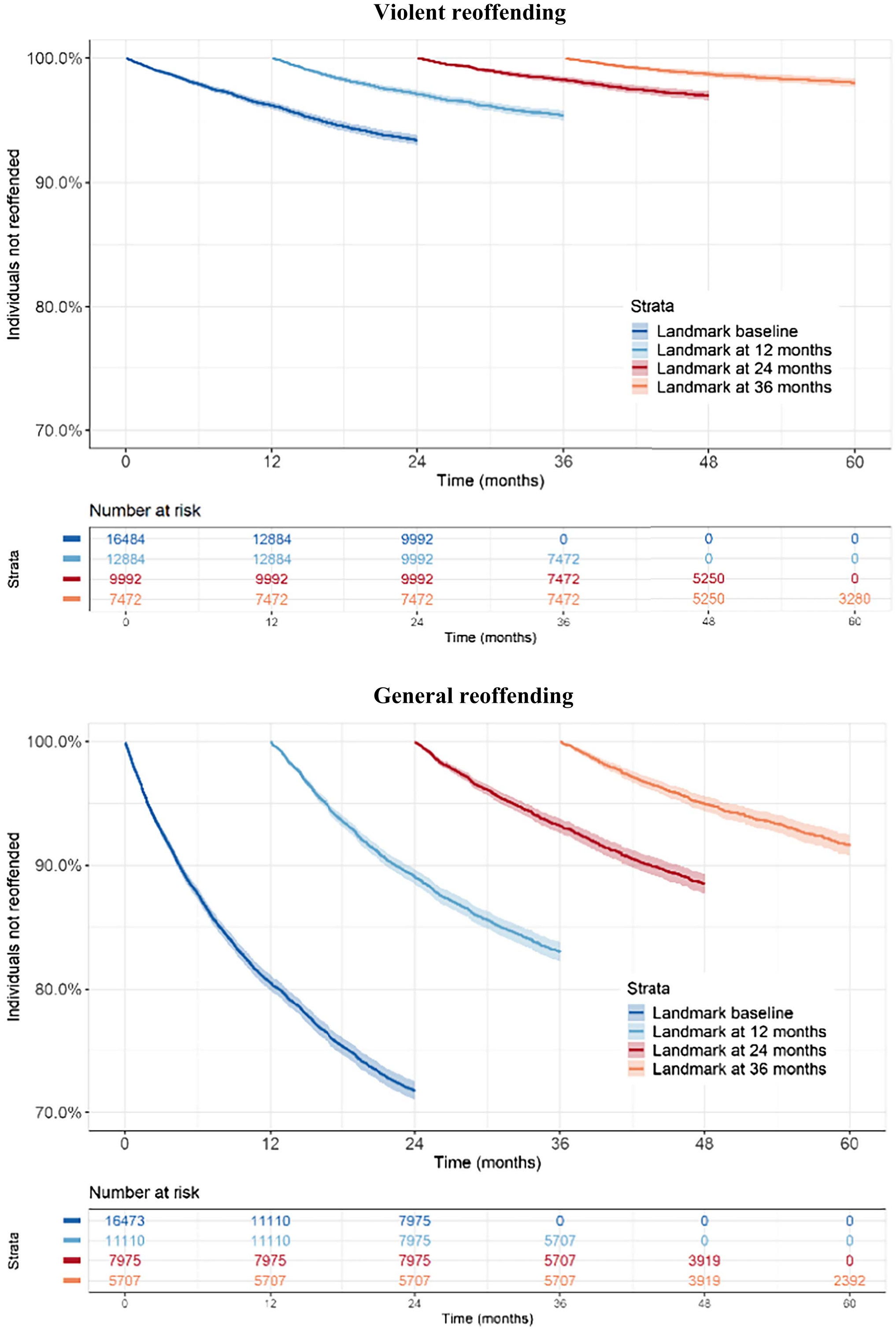

Note. See the online article for the color version of this figure.

(*Appendices continue*)

Appendix I

Receiver Operating Characteristics Curves for Internal and External Validation

Figure I1

Receiver Operating Characteristic Curves for Derivation and External Validation of the 2-Year Violent Reoffending Model at Sequential Landmark Time Points

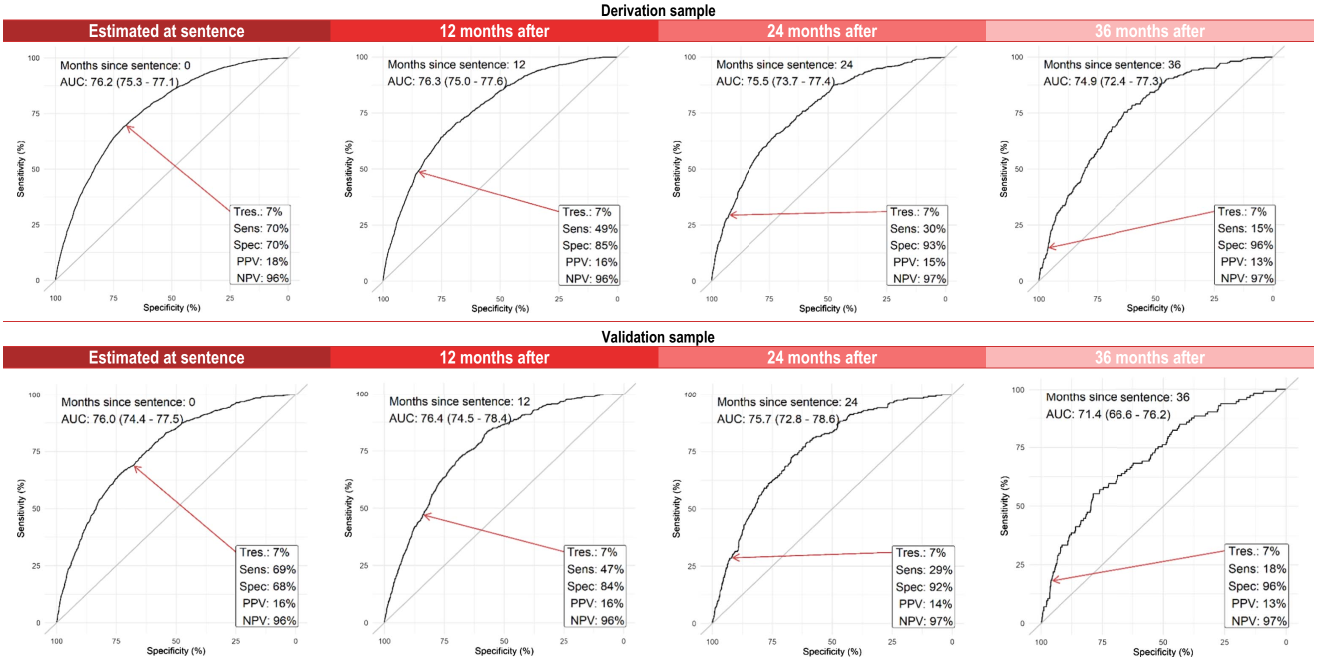

Note. Estimates for a given threshold. Tres. = threshold; Sens = sensitivity; Spec = specificity; AUC = area under curve; PPV = positive predictive value; NPV = negative predictive value. See the online article for the color version of this figure.

(*Appendices continue*)

Figure I2

Receiver Operating Characteristic Curves for Derivation and External Validation of the 2-Year General Reoffending Model at Sequential Landmark Time Points

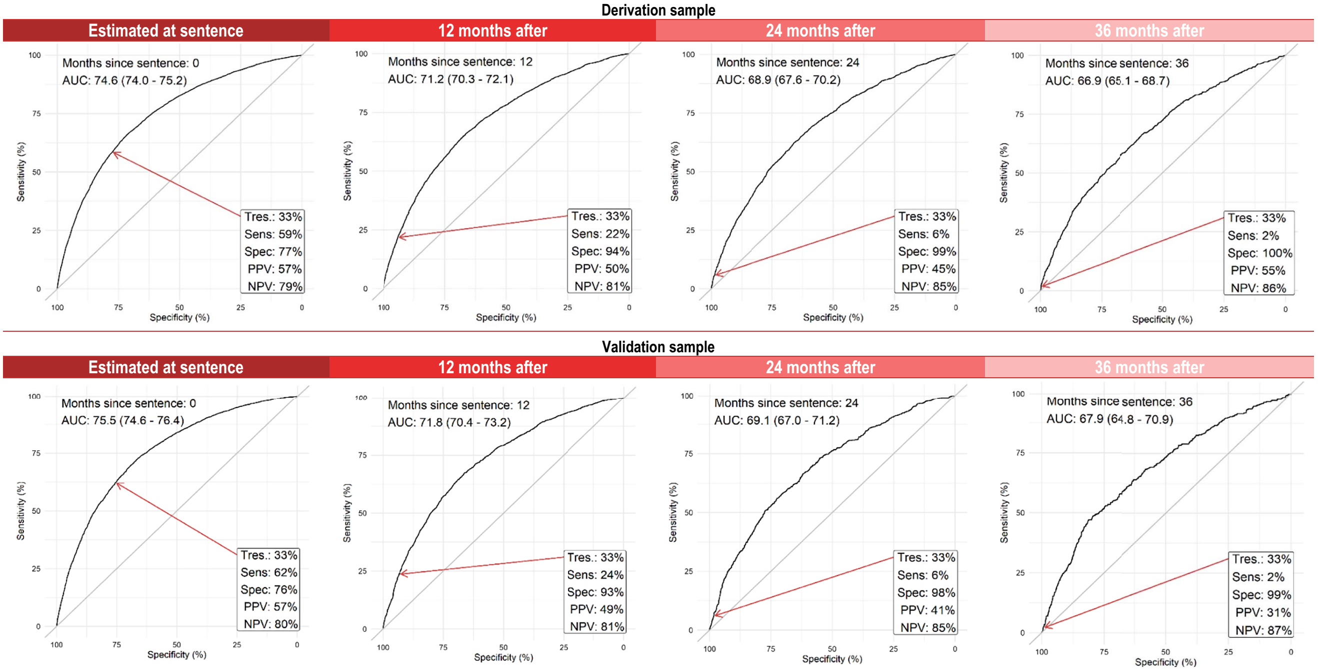

Note. Estimates for a given threshold. Tres. = threshold; Sens = sensitivity; Spec = specificity; AUC = area under curve; PPV = positive predictive value; NPV = negative predictive value. See the online article for the color version of this figure.

Appendix J

Hypothetical Vignettes

We applied the final DLM to simulated data sets containing information about hypothetical individuals given community sentences. The individuals were being supervised in the community for 3 years since the start of their sentence. Their probation officer conducted regular monthly assessments. The resulting vignettes illustrate a practical application of the developed criminal recidivism risk monitoring tool.

High Risk

Vlad is 24 y.o. (at the start of the community supervision) unemployed man with a prior criminal history. He had been imprisoned for a violent offense in the past. His index offense was nonviolent. He was diagnosed with bipolar disorder and he also experienced several self-harm episodes before the current sentence.

During month six of his sentence, Vlad got involved in the fight at the local bar. He was assaulted and injured. During month 13, he was hospitalized with substance intoxication and received an alcohol use diagnosis. During month 25, he was hospitalized with a manic episode with psychotic features for 2 weeks.

Despite his struggles, Vlad managed to remain offense-free and found a stable job by the end of his community sentence.

Low Risk

Anastasia is 35 y.o. (at the start of the community supervision) an employed woman without a prior criminal history. She is married. Her index offense was violent. She was diagnosed with paranoid schizophrenia several years before the index sentence.

During month four of her sentence, she was hospitalized with a psychotic episode and spent 1 week in a hospital. During month 24, she lost her job and was not able to find a new one. She was granted income support 7 months later.

Medium Risk

Dmitriy is 25 y.o. (at the start of the community supervision) employed man with a prior criminal history. He received a community sentence in the past for a violent offense. His index offense was not a violent offense. In the past, he was diagnosed with schizoaffective disorder, drug use disorder, and alcohol use disorder.

Over the course of his sentence, Dmitriy experienced the same events as Anastasia did. During month four of the sentence, he was hospitalized with a psychotic episode for a week. During month 24, he lost his job and was not able to find a new one. He was granted income support 7 months later.

(*Appendices continue*)

Figure J1

Predicted Risk Trajectories for a Hypothetical High-Risk Individual

Case: Vlad

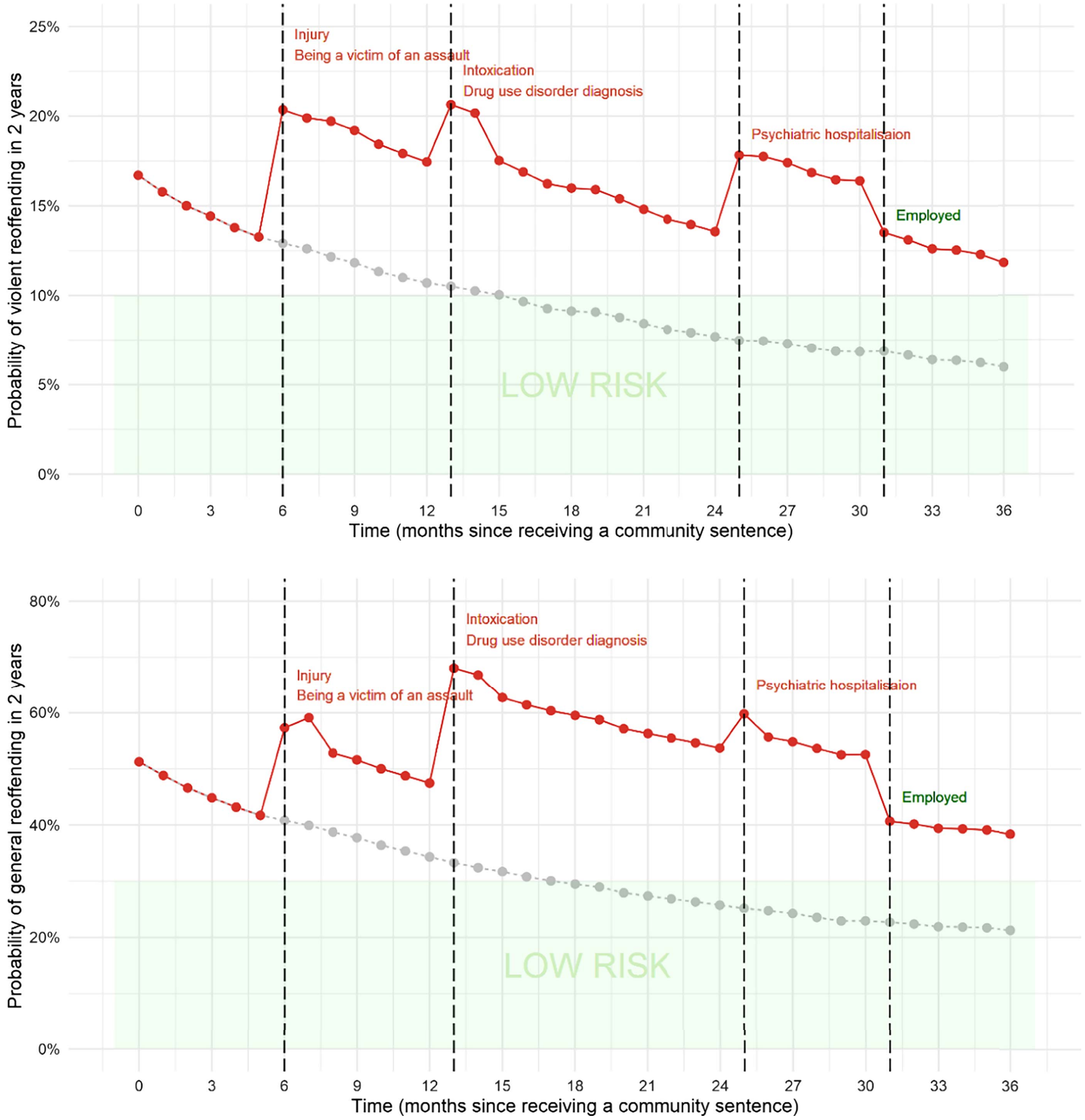

Note. Gray line represents the reoffending risk trajectory given no covariates change their values from baseline. “Low risk” area is depicted for comparison using a threshold of 30% for general reoffending and 10% for violent reoffending. See the online article for the color version of this figure.

(*Appendices continue*)

Figure J2

Predicted Risk Trajectories for a Hypothetical Low-Risk Individual

Case: Anastasia

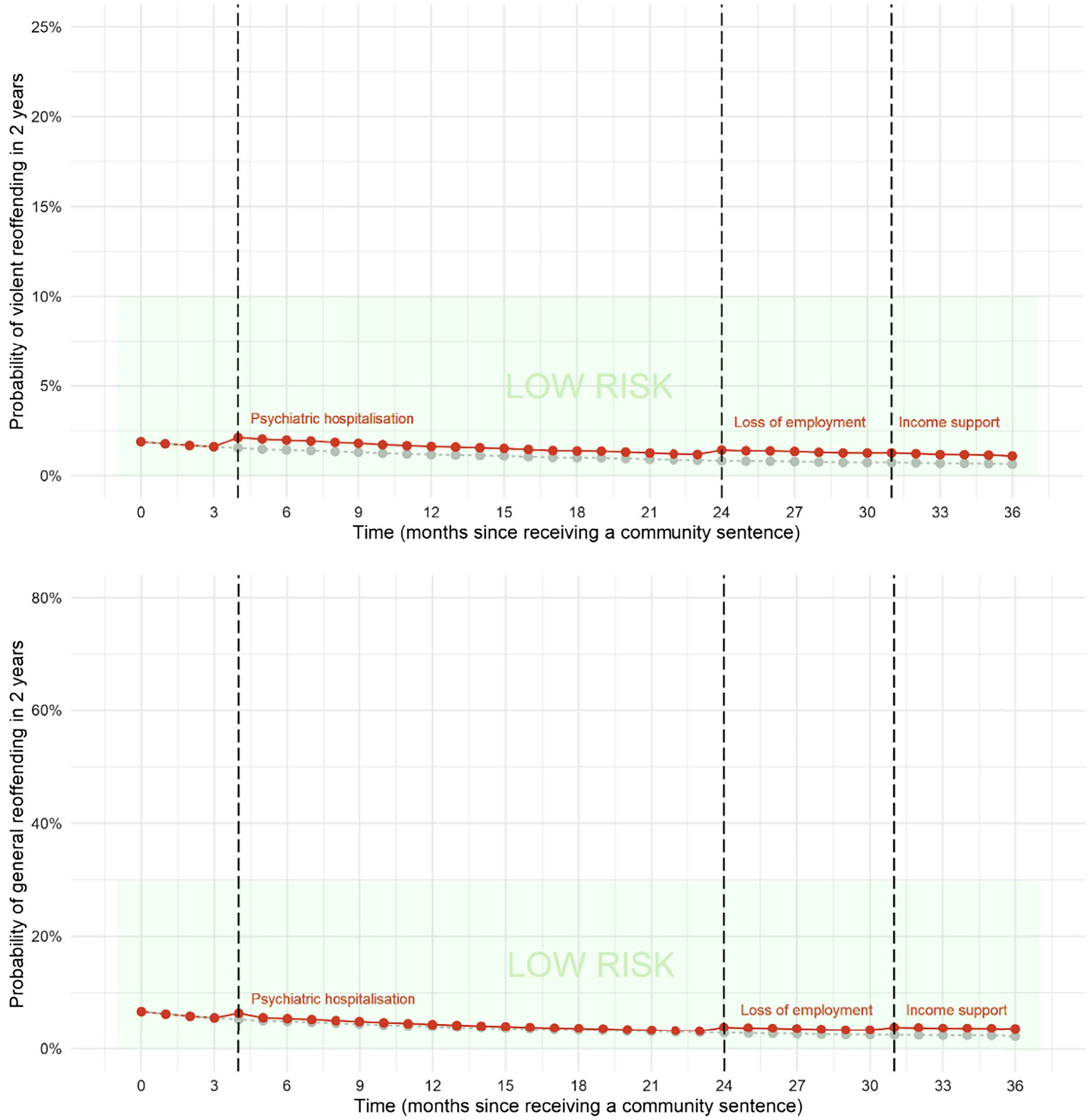

Note. Gray line represents the reoffending risk trajectory given no covariates change their values from baseline. “Low risk” area is depicted for comparison using a threshold of 30% for general reoffending and 10% for violent reoffending. See the online article for the color version of this figure.

(*Appendices continue*)

Figure J3

Predicted Risk Trajectories for a Hypothetical Medium-Risk Individual

Case: Dmitriy

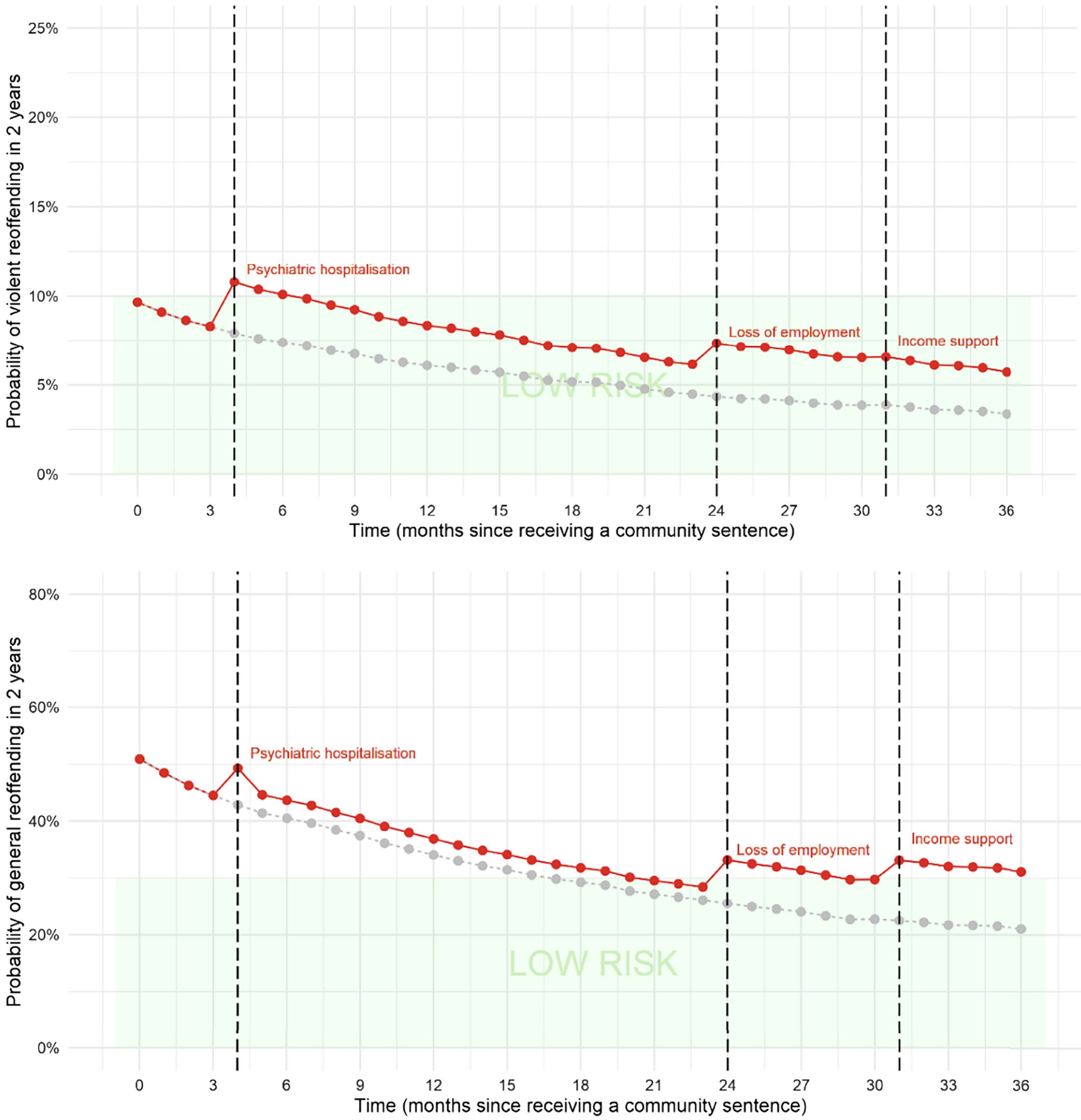

Note. Gray line represents the reoffending risk trajectory given no covariates change their values from baseline. "Low risk" area is depicted for comparison using a threshold of 30% for general reoffending and 10% for violent reoffending. See the online article for the color version of this figure.

Received November 5, 2024
Revision received September 3, 2025
Accepted September 22, 2025 ■
